# RNA methylation in urological cancers: regulatory logic, biological functions, and clinical relevance

**DOI:** 10.3389/fimmu.2026.1790638

**Published:** 2026-04-21

**Authors:** Jiahao Peng, Chong Xie, Zhangmin Xie, Feng Tang, Zhiying Wang, Minhao Liu, Li Yu, Chenhao Tang, Ziyang Liu, Zuowei Yuan, Longwei Liao, Xinpan Zou, Xuran Liu, Mingyang Yi, Huangheng Tao, Wei Wei, Tao Liu, Yixiang Liao

**Affiliations:** 1 Department of Urology, Jingzhou Hospital Affiliated to Yangtze University, Jingzhou, China; 2 Department of Urology, Jiangling People's Hospital, Jingzhou, China; 3 Department of Interventional Radiology, Jingzhou Hospital Affiliated to Yangtze University, Jingzhou, China; 4 Department of Urology, The Second Affiliated Hospital of Nanchang University, Nanchang, China; 5 Department of Gastroenterology, Qingdao Municipal Hospital, Qingdao, China; 6 State Key Laboratory of Oral & Maxillofacial Reconstruction and Regeneration, Key Laboratory of Oral Biomedicine, Ministry of Education, Hubei Key Laboratory of Stomatology, School & Hospital of Stomatology, Wuhan University, Wuhan, Hubei, China; 7 Department of Urology, Zhongnan Hospital of Wuhan University, Wuhan, China

**Keywords:** bladder cancer (BCa), N^1^-methyladenosine (m^1^A), N^6^-methylAdenosine (m^6^A), N^7^-methylguanosine (m^7^G), prostate cancer (PCa), renal cell carcinoma (RCC), RNA methylation, RNA modification

## Abstract

Urological malignancies, including prostate, bladder, and renal cancers, remain a major clinical challenge because of tumor heterogeneity, disease progression, and therapeutic resistance. In this context, RNA methylation has emerged as an important post-transcriptional regulatory layer in cancer biology. Importantly, the biological consequences of RNA methylation are not dictated by chemical modifications alone, but by coordinated networks of regulatory proteins that install, remove, and interpret these marks, thereby shaping RNA fate and gene expression programs. Among currently studied RNA modifications, N^6^-methyladenosine (m^6^A) provides the most mature mechanistic framework, whereas non-m^6^A regulators remain comparatively less well characterized. In this review, we systematically summarize the regulatory logic underlying RNA methylation-mediated control in urological cancers, with particular emphasis on how distinct classes of regulatory proteins influence RNA stability, translation, metabolism, and other post-transcriptional processes. We further integrate current evidence across multiple RNA methylation types, including m^6^A, 5-methylcytosine (m^5^C), N^1^-methyladenosine (m^1^A), and N^7^-methylguanosine (m^7^G), while highlighting the relative immaturity of non-m^6^A evidence in these malignancies. We also provide an overview of current m^6^A detection technologies and discuss their methodological strengths, limitations, and appropriate research applications. Beyond mechanistic insights, we discuss the emerging clinical relevance of RNA methylation regulators in urological malignancies, particularly in relation to diagnostic-related evidence, prognostic stratification, immune-related relevance, and treatment-associated adaptation. In addition, we summarize current therapeutic efforts targeting RNA methylation pathways, including small-molecule inhibitors and related translational strategies, while emphasizing their present limitations. Overall, current evidence supports RNA methylation as a biologically important and increasingly translationally relevant framework in urological cancers, but not yet as a clinically mature one.

## 1 Introduction

In 2022, an estimated 20 million new cancer cases were reported globally, with urological malignancies comprising roughly one-eighth of the overall cancer burden. Among these, prostate cancer is the most frequently diagnosed in men, accounting for approximately 1.47 million cases (7.3%), followed by bladder and kidney cancers at 3.1% and 2.2%, respectively. In contrast, testicular and penile cancers occur far less frequently, with incidence rates below 1% (1).

Current management strategies for urological malignancies encompass multiple therapeutic modalities, including surgical intervention, radiotherapy, chemotherapy, targeted therapy, and hormonal treatment. Among these approaches, surgery remains a cornerstone option, providing curative potential, definitive histopathological evaluation, and effective symptom control. Nevertheless, surgical management is associated with inherent limitations, such as perioperative risks, postoperative morbidity, and substantial economic burden (2–4). Depending on tumor type and stage, radiotherapy and chemotherapy may be used alongside surgery as part of multimodal treatment strategies. In prostate cancer management, brachytherapy is widely applied as a localized radiotherapy modality that enables the precise delivery of high-dose radiation to tumor tissues, thereby enhancing tumor control (5). In parallel, cisplatin-based chemotherapy plays a pivotal role in the treatment of bladder cancer, especially in metastatic settings, where it has been shown to confer substantial benefits in terms of progression-free and overall survival (6). However, traditional radiotherapy and chemotherapy are often accompanied by tissue toxicity and the development of drug resistance. In addition to their antitumor efficacy, chemotherapeutic drugs are frequently associated with treatment-related adverse effects, including gastrointestinal symptoms, alopecia, and myelosuppression, thereby markedly impairing patients’ quality of life (7–9). Targeted therapies have gained considerable attention in recent years. In parallel, since the clinical introduction of PD-1/PD-L1 immune checkpoint inhibitors in the mid-2010s, substantial progress has been achieved, particularly in the management of kidney and bladder cancers. Nevertheless, despite these advances, targeted therapies continue to face significant limitations, including the development of drug resistance, treatment-related toxicities, and considerable financial burden (10–12).

Taken together, although notable advances have been achieved in the treatment of urological malignancies, substantial challenges remain unresolved. Against this backdrop, RNA modifications—particularly N^6^-methyladenosine (m^6^A), a central post-transcriptional regulatory layer—have attracted growing interest. Accumulating evidence demonstrates that RNA methylation is intricately involved in tumor initiation and malignant progression (13).

In 1951, Cohn and Volkin reported the first modified nucleoside identified in RNA, later recognized as pseudouridine, marking the earliest described RNA modification. This discovery revealed the existence of chemical modifications within RNA molecules (14) and laid the foundation for later developments in epitranscriptomics. Epitranscriptomics refers to the investigation of chemical modifications on RNA molecules and their associated biological functions. Analogous to DNA and histone modifications in epigenetic regulation, these post-transcriptional alterations—collectively termed RNA modifications—include N^6^-methyladenosine (m^6^A), pseudouridine (ψ), 5-methylcytosine (m^5^C), and N^1^-methyladenosine (m^1^A). Through modulating RNA stability, translation efficiency, splicing patterns, subcellular localization, and degradation dynamics, these modifications exert broad regulatory effects on gene expression (15). Since the initial discovery of pseudouridine, several key developments in RNA modifications have followed. In 1974, researchers identified m^6^A, the most common mRNA modification, marking the beginning of systematic research into mRNA modifications (16). In 2011, the discovery that the FTO protein can remove m^6^A modifications revealed the reversibility and dynamic nature of RNA modifications (17). In 2012, m^6^A-seq technology was first used for large-scale mapping of m^6^A modifications, highlighting their widespread presence in mRNA and their crucial role in gene expression regulation (18). In 2016, studies demonstrated the dynamic changes of m^1^A in eukaryotic mRNA and explored its role in gene expression, sparking further research into the relationship between RNA modifications and disease (19).

RNA methylation, particularly N^6^-methyladenosine (m^6^A), has emerged as a key regulatory layer governing gene expression and has been increasingly implicated in cancer initiation and progression (20). The reversible and dynamic control of m^6^A deposition, removal, and recognition by specific “writers,” “erasers,” and “readers” provides novel avenues for cancer diagnosis and therapeutic intervention (21). Notably, aberrant expression patterns of m^6^A-related enzymes have been observed across multiple cancer types, highlighting methylation regulators as promising biomarkers for cancer diagnosis and prognosis (22).

This review systematically integrates current evidence on the regulatory mechanisms of RNA methylation–associated proteins, including N^6^-methyladenosine (m^6^A), 5-methylcytosine (m^5^C), N^1^-methyladenosine (m^1^A), and N^7^-methylguanosine (m^7^G), and delineates their functional roles in the initiation and progression of urological malignancies. We also provide an overview of current m^6^A detection technologies and discuss their methodological relevance for interpreting RNA methylation-associated findings in cancer research. In addition, we discuss the clinical relevance of these methylation regulators, including diagnostic-related evidence, prognostic stratification, immune-related relevance, and emerging therapeutic opportunities and challenges in targeting RNA methylation machinery.

## 2 RNA methylation: fundamental concepts and regulatory logic

RNA methylation constitutes a dynamic layer of post-transcriptional regulation that connects RNA processing with downstream biological outcomes. Rather than acting as isolated chemical modifications, RNA methylation functions through coordinated regulatory proteins that shape downstream RNA fate. This conceptual framework provides the basis for understanding how RNA methylation-mediated regulation operates in urological malignancies.

### 2.1 Why RNA methylation matters for urological malignancies

Urological malignancies, including prostate, bladder, and renal cancers, represent a growing clinical burden, and their management is frequently limited by therapeutic resistance, disease recurrence, and tumor heterogeneity (23). With advances in high-throughput sequencing and RNA analytical technologies, post-transcriptional regulation has emerged as a critical layer for understanding cancer-associated molecular alterations beyond DNA and transcriptional control (24). Among diverse post-transcriptional mechanisms, RNA methylation has attracted increasing attention due to its close association with tumor initiation and progression (23–25).

N^6^-methyladenosine (m^6^A) is the most extensively studied RNA methylation mark and serves as a representative model for understanding RNA-centered regulatory mechanisms in cancer (16, 26). m^6^A modification is widely distributed across multiple RNA species and occurs with defined positional preferences, supporting its capacity to exert transcriptome-wide regulatory effects (18, 27).

Importantly, the functional outcomes of RNA methylation are shaped not only by the modification itself but also by regulatory proteins that install, remove, and interpret these marks, enabling dynamic and context-dependent control of RNA fate (17, 28–30). Through modulating RNA stability, translation, splicing, and degradation, RNA methylation provides a mechanistic bridge linking molecular regulation to oncogenic phenotypes (23, 24). Together, these features establish RNA methylation as a biologically relevant regulatory layer for investigating mechanisms underlying urological tumor development and treatment-related adaptation (23).

### 2.2 Scope and fundamental concepts of RNA methylation discussed in this review

RNA methylation refers to a class of post-transcriptional chemical modifications occurring on RNA molecules, in which methyl groups are covalently added to specific sites on RNA and modulate RNA metabolism without altering the underlying nucleotide sequence (16). Compared with DNA methylation, RNA methylation is more often discussed in terms of dynamic, reversible, and context-dependent regulation, enabling relatively rapid and flexible modulation of gene expression in response to developmental cues and environmental changes (24). This regulatory plasticity renders RNA methylation particularly relevant to cancer biology, where adaptive transcriptional and translational programs are essential for tumor initiation and progression (23). To date, multiple forms of RNA methylation have been identified, among which N^6^-methyladenosine (m^6^A) is the most extensively studied and best characterized (16). In addition to m^6^A, other prevalent RNA methylation types include N^1^-methyladenosine (m^1^A), 5-methylcytosine (m^5^C), and N^7^-methylguanosine (m^7^G), each possessing distinct chemical properties and regulatory features (25, 31). Although these modifications differ in abundance, distribution, and functional output, they collectively contribute to post-transcriptional gene regulation across diverse biological contexts (32).

**Figure 1:**
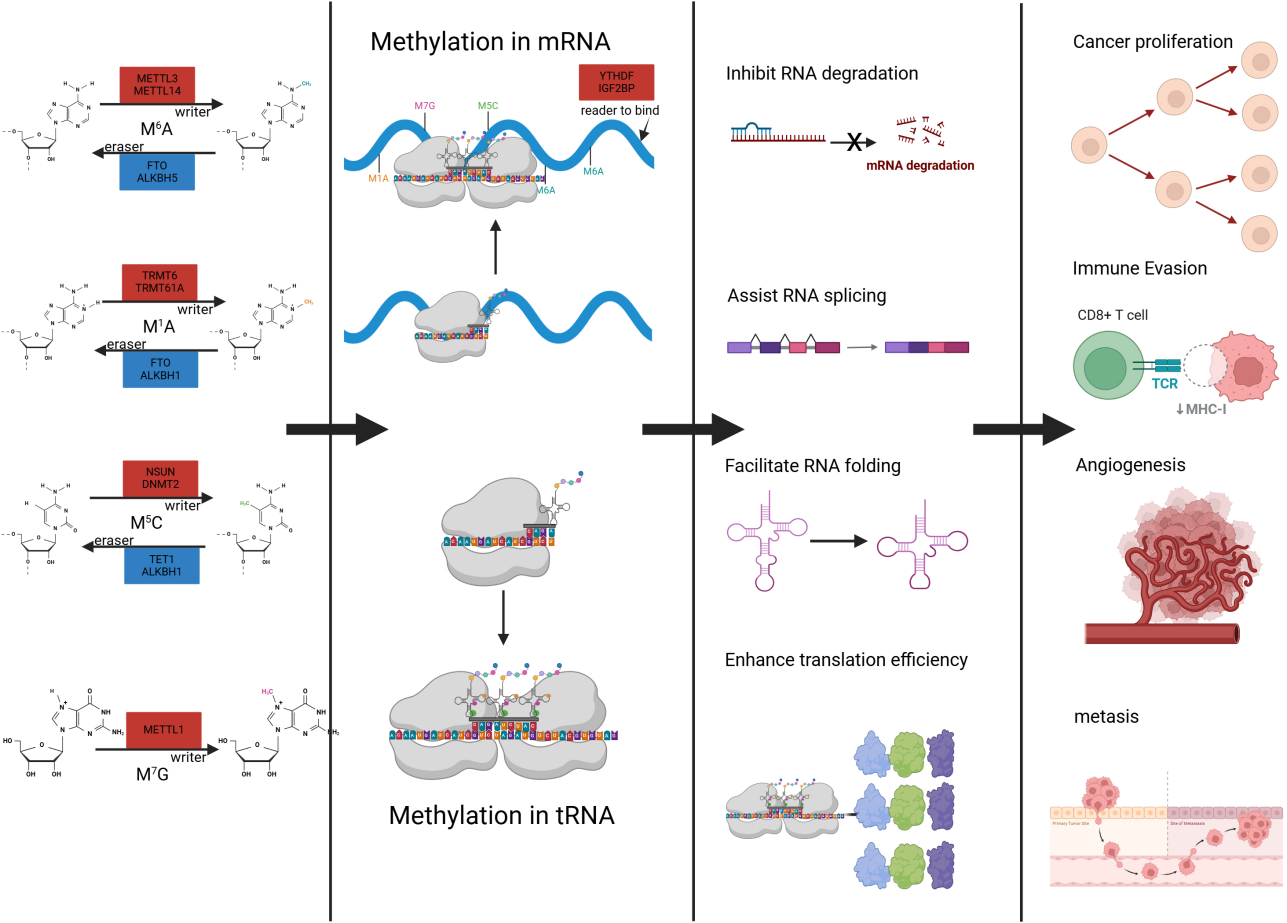
Schematic overview of RNA methylation dynamics, including m^6^A, m^1^A, m^5^C, and m^7^G, mediated by RNA writers, erasers, and readers. The figure summarizes how these modifications act on mRNA and tRNA, the resulting structural and functional consequences, and the tumor-related biological behaviors associated with dysregulated RNA methylation in urological malignancies. (This figure was created with BioRender.com).

**Figure 2:**
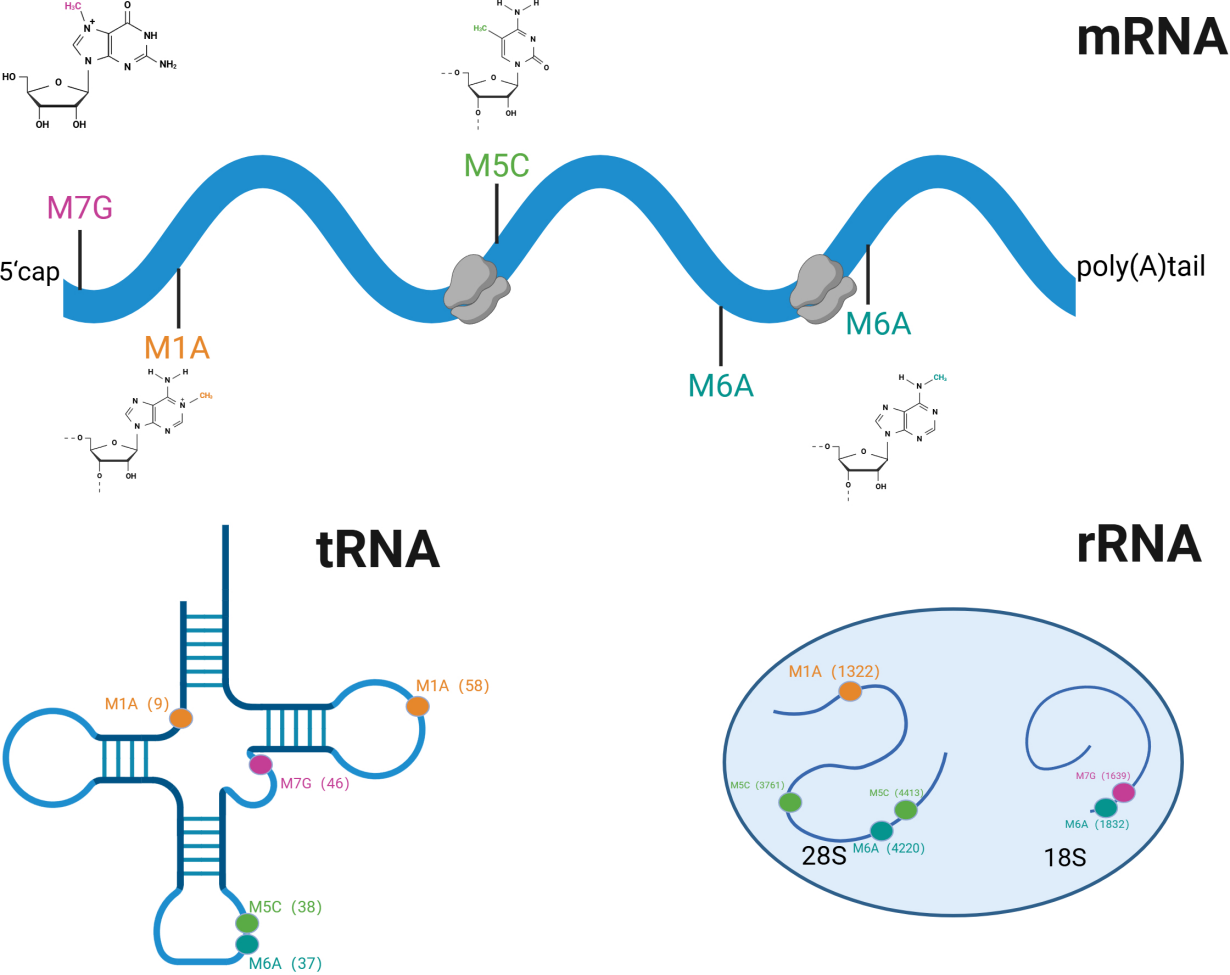
Schematic representation of the distribution of m^7^G, m^1^A, m^5^C, and m^6^A methylation modifications on mRNA, tRNA, and rRNA subunits (28S, 18S). (This figure was created with BioRender.com).

A unifying feature of RNA methylation regulation is its reliance on three functional classes of regulatory proteins: methyltransferases that deposit methyl groups (“writers”), demethylases that remove these modifications (“erasers”), and binding proteins that recognize methylated sites and mediate downstream effects (“readers”) (29). These regulators function in a coordinated manner to shape RNA fate, including transcript stability, splicing, nuclear export, translation efficiency, and degradation (24, 33). Consequently, the biological impact of RNA methylation is dictated not merely by the presence of a chemical modification but by the dynamic interplay among its regulatory proteins (30). This coordinated writer–eraser–reader regulatory framework is summarized in **Figure 1**. RNA methylation occurs across multiple RNA species, extending beyond messenger RNA to include transfer RNA, ribosomal RNA, and several classes of non-coding RNAs (25, 27). The distribution of major RNA methylation modifications across different RNA species is illustrated in **Figure 2**. Based on this framework, the present review considers RNA methylation as a coordinated regulatory system and uses this conceptual model to examine its roles in urological malignancies in subsequent sections (24).

### 2.3 Core regulatory logic of RNA methylation: how chemical marks are translated into functional outcomes

RNA methylation exerts its regulatory functions through a coordinated writer–eraser–reader framework, which helps explain how chemical RNA modifications are translated into effects on gene expression (29). Within this framework, methyltransferases (“writers”) install specific methyl marks on target RNA molecules, demethylases (“erasers”) remove these modifications, and binding proteins (“readers”) recognize methylated sites to execute downstream regulatory effects (24, 29, 30, 33). Importantly, this tripartite system operates as an integrated functional unit rather than as independent components, ensuring both regulatory specificity and dynamic responsiveness (30).

The writing process contributes to the spatial selectivity of RNA methylation by helping define which RNA species and transcript regions are modified (18). This selectivity is not random but is shaped by the coordinated action of methyltransferase complexes, which preferentially direct methyl group deposition toward particular sequence motifs and structural contexts (34–36). Through this mechanism, RNA methylation can preferentially target specific transcripts or regions, thereby shaping the regulatory potential of modified RNAs at the molecular level (18). Following methylation, reader proteins decode these chemical marks and translate them into functional RNA outputs (33). By selectively binding to methylated RNA sites, readers influence multiple aspects of RNA fate, including transcript stability, splicing patterns, nuclear export, translational efficiency, and degradation (24, 33, 37–39). Notably, distinct reader proteins can direct divergent outcomes from the same modification, helping to explain context-dependent and sometimes contrasting biological effects (28, 40).

The erasing process introduces temporal flexibility into RNA methylation regulation by enabling the removal of methyl marks in a reversible manner (17, 30). Through demethylation, RNA modifications can be dynamically adjusted in response to cellular signals, environmental stress, or developmental cues, thereby conferring plasticity to post-transcriptional gene regulation (24). This reversibility supports the view of RNA methylation as a responsive and adaptable regulatory layer rather than a purely static chemical modification (23). Collectively, the coordinated actions of writers, readers, and erasers converge on the regulation of RNA fate as a common functional endpoint (24). By modulating RNA stability, processing, localization, and translational output, RNA methylation provides a direct molecular route from chemical modification to gene expression control (23, 24).

Among known RNA methylation types, N^6^-methyladenosine (m^6^A) represents the most extensively characterized regulatory paradigm and serves as a conceptual model for understanding RNA methylation mechanisms (16). Although other RNA methylation modifications may follow partially analogous regulatory principles, m^6^A has provided the clearest insights into how writer–eraser–reader coordination translates chemical marking into functional outcomes (24). Accordingly, m^6^A is used in this review as a representative framework to illustrate core regulatory principles. This conceptual foundation provides the basis for the subsequent analysis of RNA methylation regulators and their cancer-specific roles in urological malignancies.

## 3 Methodological overview of m^6^A detection technologies

The biological interpretation of m^6^A depends fundamentally on how it is measured. Different detection platforms do not simply vary in technical sensitivity, but define distinct analytical layers of information, including global abundance, transcriptome-wide mapping, single-base resolution, native transcript context, and cellular heterogeneity. Recent methodological reviews emphasize that no single approach can simultaneously provide high sensitivity, high specificity, transcript-level assignment, stoichiometric quantification, and broad compatibility across diverse sample types (22, 41, 42). Accordingly, current m^6^A detection technologies are better understood as a complementary methodological continuum rather than as interchangeable tools. In this section, we therefore organize the major platforms according to their analytical progression—from global quantification and antibody-based transcriptome mapping to antibody-independent base-resolution strategies, direct RNA sequencing, and emerging single-cell and spatial epitranscriptomic approaches—while emphasizing their representative strengths, key limitations, and appropriate research scenarios (22, 41, 42). These representative technologies, together with their strengths, limitations, and recommended research scenarios, are summarized in **Table 1**.

**Table 1:** Comparative overview of major m^6^A detection technologies.

Technology category	Methods	Resolution/output level	Core strengths	Key limitations	Recommended research scenarios
Global Quantification	LC–MS/MS	Global/bulk-level abundance	High accuracy; high sensitivity; robust bulk comparison	No transcript assignment; no site-level information	Global m^6^A burden assessment; cohort comparison; bulk validation
Antibody-based Transcriptome Mapping	m^6^A-seq/MeRIP-seq; miCLIP	Transcriptome-wide peak mapping; higher-resolution site localization in selected methods	Established screening; scalable profiling; foundational transcriptome-wide mapping	Antibody dependence; limited resolution in enrichment-based peak methods; non-specific binding and antibody-specificity bias	Discovery-stage mapping; candidate region identification; antibody-based follow-up
Antibody-independent Base-resolution Approaches	DART-seq; GLORI; m^6^A-SAC-seq	Higher-resolution, site-specific interrogation; quantitative readout in selected methods	Antibody-free profiling; improved specificity; quantitative potential; low-input applicability in selected methods	Method-specific bias; motif dependence; calibration requirements; limited isoform context	Site validation; stoichiometry analysis; mechanistic studies; limited-input applications
Direct RNA Sequencing	Nanopore direct RNA sequencing; computational calling frameworks (e.g., Xron)	Direct single-molecule readout; long-read native RNA context; isoform-aware transcript analysis	Native RNA preservation; no cDNA conversion; long-read context; reduced isoform ambiguity	Higher error rates; sequencing cost; RNA quality requirements; computational dependence	Native transcript analysis; isoform-aware interrogation; direct signal-based m^6^A profiling
Emerging Single-cell Epitranscriptomic Methods	scDART-seq; scm^6^A-seq	Single-cell profiling; cell-specific signatures; cellular heterogeneity analysis	Resolves cellular heterogeneity; identifies cell-specific programs; links modification states to cellular context	Early-stage development; low coverage; high cost; substantial technical complexity	Heterogeneous tissues; subpopulation analysis; developmental studies; exploratory applications

### 3.1 Global quantification

Global quantification represents the most fundamental layer of m^6^A detection technologies, focusing on whether the overall abundance of RNA modification differs across samples rather than on the identity of individual modified transcripts or sites. As a representative platform in this category, LC–MS/MS has been recognized as a highly accurate and sensitive approach for the detection and quantification of modified ribonucleosides in RNA (43) and is widely used for measuring total m^6^A levels in mRNA and distinguishing nucleosides with different chemical modifications (42). Recent methodological work has further emphasized that the analytical strength of LC–MS/MS lies not only in robust quantification, but also in the standardization and reproducibility of modified ribonucleoside analysis across laboratories. From the perspective of the broader technology landscape, such biochemical quantification strategies provide an essential entry point for assessing global RNA modification burden before moving toward transcript-resolved approaches (41). However, the output of this category remains limited to bulk-level abundance information: although LC–MS/MS can sensitively capture overall changes in modification levels, it does not directly assign m^6^A signals to specific transcripts or nucleotide positions (42). This inherent limitation helps explain why subsequent technologies were developed to progress from total abundance measurement toward transcriptome-wide mapping and, ultimately, higher-resolution site interrogation.

### 3.2 Antibody-based transcriptome mapping

Antibody-based transcriptome mapping marks the transition of m^6^A detection from global abundance measurement to transcriptome-wide mapping. The pioneering m^6^A-seq and MeRIP-seq strategies, introduced independently in 2012, combined anti-m^6^A immunoprecipitation with high-throughput sequencing and thereby enabled the first transcriptome-wide maps of mammalian m^6^A methylation (18, 44). These studies showed that m^6^A is a widespread internal mRNA modification and revealed its characteristic enrichment near stop codons and within 3′ UTRs (18, 44). From a methodological perspective, this class of approaches established antibody-based enrichment plus next-generation sequencing as the foundational route for transcriptome-wide RNA modification profiling (41).

However, the original MeRIP-seq/m^6^A-seq framework generally identifies methylated regions as peaks of approximately 50–200 nt rather than precise modified nucleotides, and its performance is influenced by input RNA amount, non-specific binding and antibody-specificity bias (41, 45). In particular, m^6^A antibodies can also detect the cap-adjacent modification m^6^Am, and antibody specificity can vary substantially across sequence contexts, introducing technical bias into differential analyses (45, 46). To address the low resolution and high background of conventional peak-based enrichment, UV-crosslinking-based CLIP strategies such as miCLIP were subsequently developed within the same antibody-dependent route, allowing higher-resolution mapping, approaching single-base resolution of m^6^A sites through crosslinking-induced reverse-transcription signatures (42). Accordingly, antibody-based methods remain highly valuable for transcriptome-wide screening and discovery-oriented mapping, but their dependence on antibody performance, limited quantitative precision, and residual technical bias also explain why later technologies moved toward antibody-independent and base-resolution approaches (41, 45, 46).

### 3.3 Antibody-independent base-resolution approaches

Antibody-independent base-resolution approaches emerged in response to a central limitation of antibody-based transcriptome mapping: although MeRIP-seq and related CLIP-based methods enabled transcriptome-wide discovery, they remained constrained by antibody dependence and were not ideal for accurate site-specific quantification. As summarized in recent reviews, this second category of sequencing-based methods relies instead on enzymes, SAM analogs, or chemical reactions to improve specificity and to move m^6^A detection toward higher-resolution, site-specific interrogation and, in selected methods, quantitative readout (41, 42). Among the representative enzyme-assisted strategies, DART-seq provides an antibody-free route for transcriptome-wide m^6^A detection by fusing the cytidine deaminase APOBEC1 to an m^6^A-binding YTH domain, thereby converting methylation-adjacent sequence information into detectable C-to-U editing signatures in standard RNA-seq (47). Importantly, DART-seq can be applied with as little as 10 ng of total RNA, making it attractive for limited-input samples (47). At the same time, however, the current DART-seq workflow relies on cellular expression of APOBEC1-YTH, and overexpression of this fusion protein may not be feasible or desirable in some settings, which limits its universal applicability (42).

A further step in this antibody-independent trajectory was the development of methods that combine single-base-resolution mapping with site-specific quantitative or stoichiometric measurement. In this context, m^6^A-SAC-seq was designed as a base-resolution, quantitative whole-transcriptome sequencing strategy with calibration-standard-adjusted quantification and broad sample applicability, including low-input and archived materials (48). Similarly, GLORI uses glyoxal- and nitrite-mediated deamination to distinguish unmethylated adenosines from intact m^6^A, thereby enabling absolute quantitative transcriptome-wide m^6^A profiling at single-base resolution (49). Reviews of the field accordingly place GLORI and m^6^A-SAC-seq among the principal non-antibody approaches capable of stoichiometric measurement in addition to single-base localization (42). Nevertheless, these higher-resolution gains do not eliminate all technical trade-offs. According to the uploaded review, m^6^A-SAC-seq shows detection bias toward GAC sites and its quantification relies on a spike-in-derived standard curve, while GLORI, like most second-generation sequencing-based methods, still requires *in vitro* fragmentation and chemical or enzymatic treatment before library construction, which compromises isoform-specific information (42). Therefore, antibody-independent base-resolution approaches substantially advance the field by improving specificity, resolution, and quantitative interpretability relative to antibody-based enrichment, but they still do not fully preserve native transcript information. This unresolved gap helps explain why subsequent technological development moved toward direct RNA sequencing of original RNA molecules (42).

### 3.4 Direct RNA sequencing

Direct RNA sequencing represents a further methodological shift in m^6^A detection, moving beyond cDNA-based, chemical-assisted, or enzyme-assisted strategies toward the direct interrogation of native RNA molecules. In contrast to second-generation sequencing workflows that require reverse transcription or amplification, nanopore-based direct RNA sequencing preserves endogenous RNA modification information while providing long-read, single-molecule data with isoform-level context (41, 50). Building on this principle, Lorenz et al. showed that direct RNA nanopore sequencing can enable m^6^A detection in endogenous transcript isoforms at base-specific resolution, highlighting its potential to reduce isoform ambiguity and simplify the experimental pipeline relative to conventional indirect approaches (50). Methodologically, m^6^A calling in this framework relies on the interpretation of modification-associated perturbations in nanopore output, including raw ionic current deviations and base-calling error patterns, rather than antibody enrichment or reverse-transcription signatures (41, 51).

The computational dimension of this platform has consequently become central to its performance. For example, Xron was developed as a semisupervised framework for detecting methylated bases from raw nanopore signals, using training and fine-tuning procedures based on synthetic and immunoprecipitation-derived datasets (51). At the same time, systematic benchmarking of ten tools for m^6^A mapping from nanopore direct RNA sequencing showed that most methods exhibit a trade-off between precision and recall, that performance can improve when integrating results from multiple tools, and that negative controls help reduce intrinsic bias (52). The same benchmarking study further showed that detection capability varies across sequence motifs, while quantitative estimates of m^6^A stoichiometry can differ substantially among tools (52). These observations indicate that, although direct RNA sequencing offers a unique route toward native, long-read, and potentially isoform-aware m^6^A profiling, its analytical robustness still depends heavily on computational design and benchmarking.

From the perspective of biological application, this platform has already demonstrated value in cancer-associated transcriptome analysis. In non-small cell lung cancer (NSCLC), direct RNA nanopore sequencing was used to compare tumor and adjacent normal tissues, revealing distinct differential m^6^A patterns, reduced m^6^A density in tumors, and a set of prognostically relevant m^6^A-associated genes (53). Nevertheless, current direct RNA sequencing approaches still face important practical and analytical limitations. Recent reviews emphasize that nanopore-based m^6^A detection remains constrained by relatively high error rates, sequencing cost, RNA quality requirements, limited throughput, and inconsistencies in quantitative performance across tools (41). Accordingly, direct RNA sequencing should be viewed not as the definitive endpoint of m^6^A detection technology, but as a powerful native-RNA and isoform-aware framework that complements earlier sequencing-based approaches while still requiring further optimization in accuracy, standardization, and scalability (41, 52). This continuing need for greater sensitivity and finer biological resolution also helps explain the emergence of subsequent single-cell and spatial epitranscriptomic approaches.

### 3.5 Emerging single-cell and epitranscriptomic approaches

Emerging single-cell epitranscriptomic approaches extend m^6^A analysis beyond population-averaged measurements toward the direct interrogation of cell-to-cell heterogeneity. This shift is important because conventional transcriptome-wide profiling in bulk samples captures only an average signal across large cell populations, potentially obscuring biologically meaningful differences between individual cells (54, 55). Among the currently available strategies, scDART-seq and scm^6^A-seq represent two important early examples of single-cell m^6^A profiling. scDART-seq was developed as the first method for transcriptome-wide identification of m^6^A sites in single cells and revealed substantial heterogeneity in both the presence and abundance of m^6^A sites across individual cells; notably, it also showed that cellular subpopulations can be distinguished on the basis of RNA methylation signatures independent of gene expression alone (54). In parallel, scm^6^A-seq enabled simultaneous profiling of the m^6^A methylome and transcriptome in single oocytes/blastomeres, thereby supporting the analysis of dynamic m^6^A landscapes at single-cell resolution and providing a useful framework for studying developmental transitions and other highly dynamic biological states (56). Together, these studies indicate that m^6^A research is beginning to move from bulk-level mapping toward the dissection of methylation heterogeneity, dynamic regulation, and cell-state-specific epitranscriptomic organization. At the same time, this field remains clearly emergent rather than mature: current single-cell epitranscriptomic technologies are still in an early stage of development, with limited modification coverage, restricted applicability across sample types, and continuing challenges related to throughput, coverage, standardization, and data sparsity/dropout (55). In this context, spatially resolved epitranscriptomic strategies constitute an important frontier with the potential to link RNA modification states to tissue architecture and microenvironmental context. However, they are currently better regarded as a forward-looking extension of the field than as an established methodological mainstay. Accordingly, their representative strengths, limitations, and recommended research scenarios are more appropriately summarized in the comparative table.

### 3.6 Integrated perspective on m^6^A detection strategies

Taken together, current m^6^A detection technologies should be viewed as a multilayered toolkit rather than a linear hierarchy in which one platform universally supersedes another. Sequencing-independent biochemical methods remain important for global quantification, antibody-based approaches are still valuable for transcriptome-wide screening, non-antibody methods have improved site-specific resolution and quantitative interpretation, and direct RNA sequencing offers unique advantages for native and isoform-aware interrogation (22, 41, 42). Emerging single-cell epitranscriptomic strategies further extend this framework toward cellular heterogeneity, while spatially resolved epitranscriptomic approaches remain an important but still technically immature frontier (22, 41, 42). Therefore, the selection of an m^6^A detection strategy should be driven by the biological question, the required level of resolution, and sample constraints, rather than by the assumption that a single technology can satisfy all analytical demands. Future progress in the field will likely depend on integrating complementary platforms to achieve greater accuracy, quantitative robustness, and contextual resolution in epitranscriptomic analysis (22, 41, 42).

## 4 m^6^A methylation regulators in prostate cancer

Among urological malignancies, prostate cancer (PCa) currently has a relatively substantial body of evidence concerning m^6^A regulators. Although the reported downstream mechanisms are diverse, their biological consequences tend to converge on a limited set of malignant phenotypes, most notably sustained proliferation, invasion and metastasis, stemness, metabolic adaptation, and treatment-related plasticity (57–64). Within this landscape, the writer layer is largely dominated by METTL3-centered mechanisms whose functional consequences in cancer are predominantly pro-tumor, whereas the principal eraser-associated mechanisms identified so far, particularly those involving FTO and ALKBH5, more often show tumor-suppressive consequences (63–76). By contrast, the reader layer appears broader and somewhat more heterogeneous: mechanisms involving YTHDF1, YTHDF2, IGF2BP2, and IGF2BP3 are more often linked to malignant progression, whereas the IGF2BP family has also been implicated in a distinct R-loop–associated setting with tumor-suppressive functional consequences (77–84). Overall, current evidence suggests that m^6^A regulation in PCa is better viewed as a convergent regulatory network than as a set of isolated pathways, while mechanisms involving selected regulators still display context-dependent heterogeneity (57–84). Representative m6A regulators and their associated mechanisms in PCa are summarized in **Table 2**.

**Table 2:** Representative m^6^A regulators and associated mechanisms in PCa.

m^6^A regulators	Roles in m^6^A	Molecular mechanism	Functional consequence of the mechanism in cancer	Ref
METTL3	Writer	METTL3/DGCR8/miR-182	Promotes tumor proliferation, migration, invasion, and tumorigenesis	(57)
METTL3	Writer	METTL3/PVT1/miR-27b-3p/BLM	Promotes tumor proliferation, migration, and invasion	(58)
METTL3	Writer	miR-320d/METTL3/KIF3C	Promotes tumor progression, migration, and invasion	(59)
METTL3	Writer	METTL3/circRBM33/FMR1/PDHA1	Promotes tumor growth and invasion	(60)
METTL3	Writer	METTL3/circGLIS3/miR-661/MDM2	Promotes tumor proliferation, migration, and invasion	(61)
METTL3/IGF2BP2	Writer/Reader	METTL3/circABCC4/IGF2BP2/CCAR1/Wnt/β-catenin	Promotes tumor stemness and metastasis	(62)
METTL3	Writer	METTL3/lnc-SNHG7/SRSF1/c-Myc/glycolysis	Promotes tumor initiation, progression, and glycolytic reprogramming	(63)
METTL3/IGF2BP2	Writer/Reader	METTL3/lnc-PCAT6/IGF2BP2/IGF1R	Promotes bone metastasis and tumor growth	(64)
METTL3	Writer	METTL3/lnc-MALAT1/PI3K/AKT	Promotes tumorigenesis and progression	(65)
METTL3/YTHDF2	Writer/Reader	METTL3/YTHDF2/LHPP/NKX3-1/AKT	Promotes tumorigenesis and progression	(66)
METTL3	Writer	METTL3/MYC	Promotes tumorigenesis and progression	(67)
METTL14/YTHDF2	Writer/Reader	METTL14/THBS1/YTHDF2	Promotes tumorigenesis and progression	(68)
METTL14	Writer	METTL14/CDK4/FOXM1/ATG7	Inhibits tumor invasion and migration	(69)
ALKBH5	Eraser	ALKBH5/TSPAN1	Inhibits tumor progression and autophagy	(70)
FTO/IGF2BP2,3	Eraser/Reader	FTO/DDIT4/IGF2BP2,3	Inhibits EMT-related plasticity, migration, and invasion	(71)
FTO	Eraser	FTO/MC4R	Inhibits tumor progression and migration	(72)
FTO	Eraser	FTO/CLIC4	Inhibits tumor progression and migration	(73)
IGF2BPs	Reader	IGF2BPs/SEMA3F/DNMT1	Inhibits tumor migration and induces growth retardation	(77)
IGF2BP2	Reader	IGF2BP2/HOXC6	Promotes tumorigenesis and progression	(78)
IGF2BP3	Reader	circ_0003258/IGF2BP3/HDAC4/ERK	Promotes tumor metastasis	(79)
YTHDF1	Reader	YTHDF1/TRIM44	Promotes tumor proliferation, invasion, and migration	(80)

### 4.1 m^6^A “writers” in PCa

To date, the main m^6^A writers implicated in PCa include METTL3, METTL14, and WTAP. Overall, the writer landscape in PCa is characterized by a dominant METTL3-centered pattern in which the reported downstream mechanisms more often produce pro-tumor consequences, while mechanisms involving METTL14 show clearer context dependence and those involving WTAP display context-dependent behavior mainly in circRNA-centered settings (57–69, 74–76).

Among these regulators, METTL3 is one of the most extensively studied writers in PCa, and the currently available evidence shows that METTL3-associated downstream mechanisms most often lead to pro-tumorigenic consequences. Despite the diversity of its downstream partners, the available studies point to several recurrent outputs, including enhanced proliferation and survival, increased migration and invasion, maintenance of stem-like properties, metabolic rewiring, metastatic dissemination, and treatment adaptation. At the level of miRNA-associated regulation, Wang et al (57). reported that an METTL3-associated m^6^A mechanism enhances the interaction between pri-miRNAs and DGCR8, thereby promoting pri-miR-182 processing and accelerating PCa cell proliferation. Chen et al (58). further demonstrated that a METTL3-associated ceRNA network centered on PVT1/miR-27b-3p/BLM promotes proliferation, migration, and invasion. Similarly, Ma et al (59). reported that the miR-320d/METTL3/KIF3C axis contributes to malignant progression, further supporting the view that METTL3-associated miRNA-related mechanisms can produce pro-tumor consequences in PCa.

METTL3-associated regulation also exerts important effects through circRNA-associated pathways. Zhong et al (60). showed that m^6^A-modified circRBM33 interacts with FMR1 to stabilize PDHA1 mRNA, thereby enhancing mitochondrial metabolic activity and promoting tumor growth and invasion. Cheng et al (61). demonstrated that an METTL3-associated mechanism stabilizing circGLIS3 promotes proliferation, migration, and invasion through the circGLIS3/miR-661/MDM2 pathway, while circGLIS3 silencing increases sensitivity to androgen receptor signaling inhibitors (ARSIs), including enzalutamide. In a related context, Huang et al (62). reported that an METTL3-associated m^6^A mechanism acting on circABCC4 promotes CCAR1 expression through IGF2BP2, activates Wnt/β-catenin signaling, and enhances stemness and metastatic potential. Together, these findings indicate that circRNA-centered mechanisms involving METTL3 in PCa are closely linked to metabolic adaptation, stemness, metastasis, and treatment response.

The pro-tumor functional consequences linked to METTL3-associated mechanisms in PCa further extend to lncRNA-associated networks and broader growth-promoting programs. Liu et al (63). reported that the METTL3/lnc-SNHG7/SRSF1/c-Myc/glycolysis axis promotes tumor initiation, progression, and glycolytic reprogramming. Lang et al (64). showed that METTL3-mediated m^6^A modification enhances PCAT6 expression in an IGF2BP2-dependent manner, and that the resulting PCAT6/IGF2BP2/IGF1R complex promotes bone metastasis and tumor growth. Mao et al (65). demonstrated that an METTL3-associated mechanism promotes proliferation and invasion partly through MALAT1 and PI3K/AKT signaling, whereas Li et al (66). found that a METTL3/YTHDF2-associated mechanism reduces LHPP and NKX3–1 expression, thereby enhancing AKT phosphorylation. Consistently, Yuan et al (67). linked METTL3-associated regulation to MYC-driven prostate tumorigenesis. In addition to these tumor-intrinsic effects, Jia et al (75). extended METTL3-associated regulation to the tumor immune microenvironment by showing that prostate cancer cells promote M2 macrophage polarization through an LXA4/STAT6/METTL3-related mechanism. Taken together, the current literature consistently places METTL3-associated mechanisms at the center of a writer-dominated network in PCa whose downstream programs predominantly yield pro-tumor consequences.

Compared with METTL3-associated mechanisms, those involving METTL14 show clearer context dependence in PCa. Wang et al (68). supported a context in which the METTL14-associated mechanism exerts pro-tumor consequences, reporting that METTL14 was upregulated in PCa, associated with poorer overall survival, and promoted tumor proliferation *in vitro* and *in vivo*. This conclusion was supported by tissue microarray and survival analyses, bidirectional gain- and loss-of-function experiments, xenograft assays, and combined RNA-seq/MeRIP-seq screening, which identified THBS1 as a downstream target. They further showed that METTL14-mediated m^6^A deposition facilitated YTHDF2-dependent decay of THBS1 mRNA, thereby suppressing a tumor-suppressive transcript. In contrast, Zhong et al (69). reported that METTL14 was downregulated in PCa and that the METTL14-associated mechanism in their model exerted tumor-suppressive consequences, with evidence from clinical and cellular expression analyses, overexpression-based functional assays, xenograft experiments, MeRIP-qPCR, mRNA stability analysis, and CDK4-centered rescue experiments. In that setting, METTL14 increased m^6^A modification of CDK4, reduced CDK4 mRNA stability, and attenuated the downstream FOXM1/ATG7-mediated autophagic program. These opposing findings likely reflect differences in the dominant transcript programs captured in each model, since one study centered on YTHDF2-dependent decay of the tumor-suppressive target THBS1, whereas the other emphasized destabilization of the oncogenic effector CDK4. At present, mechanisms involving METTL14 in PCa are best regarded as context-dependent, with different METTL14-associated mechanisms producing either pro-tumor or tumor-suppressive consequences depending on the dominant transcript program engaged, although the THBS1/YTHDF2-centered pro-tumor model is currently supported by a broader mechanistic framework.

WTAP-associated regulation in PCa also appears context-dependent, particularly in circRNA-centered settings. Ding et al (76). provided relatively direct evidence that WTAP-dependent m^6^A activity promotes metastatic progression in PCa, using RNA pulldown, RIP, dot blot, MeRIP-seq/MeRIP-qPCR, and functional assays to show that circPDE5A suppresses metastasis by binding WTAP, blocking WTAP-dependent m^6^A modification of EIF3C mRNA, and attenuating the downstream EIF3C/MAPK axis. In contrast, Kong et al (74). described a distinct framework in which WTAP, together with METTL3 and METTL14, promoted m^6^A-mediated biogenesis of circDDIT4, based on expression analysis, functional assays, RNA pulldown/RIP, and m^6^A-related assays showing that circDDIT4 acts as a tumor-suppressive circRNA through the ELAVL1/HuR–ANO7 pathway. These findings suggest that the apparent discrepancy may arise less from a simple contradiction in WTAP itself than from differences in the dominant transcript outputs captured in each model: in one case, WTAP-dependent m^6^A enhances an oncogenic EIF3C-centered program, whereas in the other it facilitates formation of a suppressive circRNA. At present, WTAP-associated mechanisms in PCa are therefore best described as context-dependent, with different WTAP-associated programs producing distinct functional consequences according to the dominant transcript outputs captured in a given model.

### 4.2 m^6^A “erasers” in PCa

In contrast to the predominantly pro-tumor functional consequences observed in METTL3-centered writer-associated mechanisms, the currently available evidence in PCa suggests that eraser-associated mechanisms more often show tumor-suppressive consequences. The two principal demethylase-related contexts reported so far involve ALKBH5 and FTO, both of which are downregulated in PCa, and restoration of either regulator is associated with inhibition of malignant phenotypes (70–73).

Zhao et al (70). reported that ALKBH5 expression is markedly reduced in PCa cells. Restoration of ALKBH5 expression is associated with suppression of proliferation, reduction of EdU positivity, a lower LC3B-II/LC3B-I ratio, and increased P62 expression, collectively indicating inhibition of autophagy. Mechanistically, ALKBH5 reduces m^6^A modification of TSPAN1, whereas TSPAN1 overexpression counteracts the tumor-suppressive consequences associated with ALKBH5 restoration. These findings support a model in which the ALKBH5/TSPAN1-associated mechanism restrains PCa progression by suppressing autophagy-related and proliferative programs.

Among the currently reported eraser-associated mechanisms in PCa, those involving FTO are the most consistently linked to tumor-suppressive consequences. One study (71) showed that m^6^A regulation exhibits context-dependent dynamics during epithelial–mesenchymal transition (EMT): although global m^6^A levels are elevated in tumor tissues relative to normal tissues, they decline following EMT induction, and FTO loss enhances migration, invasion, and EMT potential. In this setting, DDIT4 was identified as a key m^6^A-regulated target recognized by IGF2BP2 and IGF2BP3, suggesting that FTO-associated regulation contributes to control of EMT-related plasticity rather than merely affecting baseline proliferation. Consistently, Li et al (72). reported that FTO is significantly downregulated in PCa and that loss of FTO is associated with increased proliferation, migration, invasion, and tumor growth, at least partly through MC4R-related signaling. Similarly, Zou et al (73). confirmed that reduced FTO expression predicts poor prognosis and showed that FTO-associated regulation restrains PCa progression by limiting m^6^A-dependent destabilization of CLIC4 mRNA. Taken together, the available evidence indicates that FTO-associated mechanisms in PCa predominantly yield tumor-suppressive consequences, with recurrent effects centered on restricting proliferation, migration, invasion, EMT-related plasticity, and metastatic progression.

### 4.3 m^6^A “readers” in PCa

The reader layer in PCa is functionally more heterogeneous than the eraser layer. The main readers reported to date include IGF2BP1, IGF2BP2, IGF2BP3, YTHDF1, and YTHDF2. Overall, most available studies indicate that mechanisms involving YTHDF1, YTHDF2, IGF2BP2, and IGF2BP3 more often produce pro-tumor consequences, whereas the IGF2BP family has also been implicated in a distinct R-loop–associated setting with tumor-suppressive consequences (77–84).

Among m^6^A readers in PCa, Ying et al (77). provided a notable suppressive counterexample within the IGF2BP family. Using S9.6 IP/LC-MS/MS, RNA pull-down, KH-domain mutagenesis, DRIP-seq/RNA-seq integration, ChIP-qPCR, and *in vitro*/*in vivo* assays, the authors identified IGF2BPs as m^6^A-dependent readers of co-transcriptional R-loops rather than canonical cytoplasmic stabilizers of pro-tumor mRNAs. Mechanistically, IGF2BPs bound m^6^A-modified DNA: RNA hybrids at the SEMA3F promoter, precluded DNMT1 and YTHDF2 binding, reduced promoter methylation, and upregulated the tumor-suppressive gene SEMA3F, thereby inhibiting PCa growth and migration and enhancing docetaxel chemosensitivity. This study is best interpreted as a non-canonical, R-loop-centered exception within the PCa reader landscape, rather than as evidence that mechanisms involving the IGF2BP family uniformly produce tumor-suppressive consequences.

In contrast, He et al (78). demonstrated that IGF2BP2 directly binds and stabilizes HOXC6 mRNA, thereby reinforcing a METTL3-dependent pro-tumor signaling program and promoting proliferation, invasion, migration, stemness, and glycolysis. Yu et al (79). further showed that IGF2BP3 binds circ_0003258 in the cytoplasm, stabilizes HDAC4 mRNA, activates ERK signaling, and promotes EMT and metastasis. Taken together, these findings indicate that the dominant pattern of IGF2BP2- and IGF2BP3-associated mechanisms in PCa remains one of pro-tumor functional consequences, while the Ying et al (77). study should be retained as an important non-canonical counterexample rather than dismissed as a simple outlier.

Compared with the IGF2BP family, YTHDF1-associated mechanisms show more consistently pro-tumor functional consequences in PCa. Li et al (80). reported that YTHDF1 is highly expressed in PCa tissues and cells, that its upregulation correlates with poor prognosis, and that YTHDF1-associated regulation promotes proliferation, migration, and invasion through TRIM44. Nie et al (82). further demonstrated that a YTHDF1-associated mechanism regulates the androgen function–related gene TRIM68, thereby influencing AR expression, cell viability, apoptosis, proliferation, migration, invasion, and *in vivo* tumor growth. In addition, Li et al (83). linked YTHDF1 to PCa progression through the ELK1/YTHDF1/PLK1/PI3K/AKT axis. Taken together, current evidence indicates that YTHDF1-associated mechanisms are among the most consistently linked to pro-tumor consequences in PCa, particularly in relation to proliferative signaling, invasive behavior, and AR-associated progression.

YTHDF2-associated mechanisms are also predominantly linked to malignant progression in PCa, although the currently available evidence is relatively more concentrated in miRNA-related pathways. Li et al (84). reported that YTHDF2 and miR-493-3p participate in PCa progression by indirectly regulating m^6^A levels. Similarly, Du et al (81). found that KDM5A promotes PCa progression through the miR-495/YTHDF2/m^6^A-MOB3B axis. In addition, as noted above, YTHDF2 also participates in METTL3-dependent downregulation of LHPP and NKX3–1 in PCa. Therefore, although YTHDF2-associated mechanisms have not yet been functionally characterized to the same breadth as those involving YTHDF1, the available studies consistently place them within a reader network whose functional consequences are predominantly pro-tumor.

In summary, current evidence suggests that m^6^A regulation in PCa is organized around a dominant METTL3-centered writer network whose downstream mechanisms are predominantly associated with pro-tumor consequences, an eraser layer centered on FTO and ALKBH5 in which the reported mechanisms more often show tumor-suppressive consequences, and a reader layer in which the currently described mechanisms are largely associated with pro-tumor outcomes, albeit with limited but important context-dependent exceptions. Across these regulators, the most recurrent phenotypic outputs are sustained proliferation, invasion and metastatic dissemination, stemness, metabolic adaptation, and treatment-related plasticity. At the same time, mechanisms involving selected regulators such as METTL14, WTAP, and the IGF2BP family show non-uniform functional consequences across different transcript and regulatory contexts, indicating that the biological consequences of m^6^A regulation in PCa are shaped less by regulator identity alone than by dominant transcript outputs and downstream regulatory coupling (57–84).

## 5 m^6^A methylation regulators in bladder cancer

Compared with prostate cancer and renal cell carcinoma, the currently available evidence in bladder cancer (BCa) shows a relatively more convergent functional pattern for m^6^A regulators. Although the downstream mechanisms are diverse, the reported biological outputs repeatedly converge on sustained proliferation, invasion and migration, stemness maintenance, metabolic reprogramming, angiogenesis, and therapy-related adaptation (85–96). Within this framework, the writer layer is dominated by METTL3-centered mechanisms whose downstream programs more often produce pro-tumor consequences, with WTAP-associated mechanisms further reinforcing malignant progression through metabolic, stress-adaptive, and immune-related pathways, whereas mechanisms involving METTL14 more often show tumor-suppressive consequences (85–96). In the eraser layer, mechanisms involving ALKBH5 are mainly linked to tumor-suppressive consequences, whereas those involving FTO more consistently produce pro-tumor consequences (97–100). Among readers, mechanisms involving YTHDF1 are the most consistently linked to pro-tumor consequences, whereas mechanisms involving YTHDC1 provide an important tumor-suppressive counterpart (101–106). Overall, current evidence suggests that m^6^A regulation in BCa is best understood as a relatively coherent regulatory network in which the currently reported mechanisms more often produce pro-tumor consequences, while a limited number of other mechanisms provide tumor-suppressive counterweights, rather than as a highly contradictory system.

### 5.1 m^6^A “writers” in BCa

In BCa, the principal m^6^A writers include METTL3, METTL14, and WTAP. Overall, the writer landscape in BCa is characterized by a dominant pattern centered on METTL3- and WTAP-associated mechanisms, whose reported downstream programs more often produce pro-tumor consequences, whereas mechanisms involving METTL14 more often show tumor-suppressive consequences (85–96).

Among these regulators, METTL3 is one of the most extensively investigated writers in BCa, and the currently available evidence indicates that METTL3-associated downstream mechanisms recurrently produce pro-tumor consequences. Across currently available studies, METTL3-associated mechanisms repeatedly converge on enhanced proliferation, tumorigenesis, invasion and migration, stemness maintenance, and angiogenesis. At the level of miRNA-associated regulation, Han et al (85). demonstrated that an METTL3-associated m^6^A mechanism enhances pri-miR-221/222 processing through DGCR8, thereby promoting pro-tumor signaling in BCa. Similarly, Yan et al (86). reported that an METTL3-associated mechanism promoting pri-miR-146a-5p maturation activates the NUMB/NOTCH2 pathway and attenuates melittin-induced apoptosis, thereby conferring a survival advantage to BCa cells. These findings indicate that METTL3-associated miRNA-processing mechanisms contribute to BCa progression in part by facilitating maturation of pro-tumor miRNAs.

Beyond miRNA processing, METTL3-associated mechanisms also contribute to BCa progression through transcriptional and stemness-related regulatory programs. Cheng et al (87). identified AFF4, together with the NF-κB components IKBKB and RELA and the oncogene MYC, as direct targets of METTL3-mediated m^6^A regulation, thereby defining a METTL3/AFF4/NF-κB/MYC network that promotes bladder cancer proliferation, invasion, and survival. Consistently, Gao et al (88). showed that loss of AFF4 phenocopies METTL3 depletion and significantly impairs the tumor-initiating capacity of bladder cancer stem cells *in vivo*. Mechanistically, AFF4 directly sustains SOX2 and MYC transcription, supporting the concept that the METTL3–AFF4–SOX2/MYC axis promotes BCa stemness and tumorigenesis. Together, these studies indicate that mechanisms involving METTL3 are important contributors to BCa growth and tumor-initiating potential.

The pro-tumor consequences linked to METTL3-associated mechanisms in BCa are further reinforced through coordinated writer–reader interactions. Xie et al (89). reported that the METTL3/YTHDF2 axis promotes BCa proliferation and metastasis by selectively degrading the mRNAs of the tumor suppressors SETD7 and KLF4. Yang et al (90). similarly demonstrated that the METTL3/CDCP1/YTHDF1 signaling axis promotes BCa progression. In another study, Shen et al (91). linked the TGF-β/Smad2/3 pathway to the RBM15/METTL3 writer complex and showed that ENO1 mRNA is methylated and subsequently recognized by YTHDF1, enhancing ENO1 translation and promoting BCa proliferation and tumor growth by reducing PCNA K48-linked ubiquitination. In addition, Wang et al (92). demonstrated that an METTL3-associated m^6^A mechanism is required for activation of the TEK–VEGF-A pathway, thereby facilitating BCa proliferation and angiogenesis. Taken together, despite mechanistic diversity, the available literature consistently places METTL3-associated mechanisms at the center of a writer-dominated network in BCa whose downstream mechanisms predominantly yield pro-tumor consequences.

Compared with METTL3-associated mechanisms, those involving METTL14 have been less extensively studied in BCa, but the currently available evidence more often supports tumor-suppressive consequences. Gu et al (93). demonstrated that an METTL14-associated m^6^A mechanism reduces Notch1 mRNA stability and thereby limits activation of Notch1 signaling, which is closely linked to bladder tumorigenesis and tumor-initiating cell self-renewal. Functionally, METTL14 knockdown enhances tumor-initiating cell proliferation, self-renewal, metastasis, and tumor-initiating ability, whereas METTL14 overexpression exerts the opposite effects. These findings indicate that METTL14-associated mechanisms restrain BCa stemness and tumor initiation.

WTAP-associated mechanisms represent another important writer-related component of BCa in which the reported downstream programs more often produce pro-tumor consequences. Tan et al (94). demonstrated that a WTAP-associated mechanism enhances the stability of PIGT mRNA through IGF2BP2-mediated m^6^A regulation, thereby promoting BCa cell proliferation, glycolysis, and metastasis by modulating GLUT1 glycosylation and membrane transport. In addition to metabolic reprogramming, Wang et al (95). showed that a WTAP-associated mechanism suppresses erastin-induced ferroptosis by increasing m^6^A modification within the 3′UTR of NRF2 mRNA, thereby enhancing NRF2 stability in a YTHDF1-dependent manner. Beyond tumor-intrinsic functions, Wang et al (96). further identified an immune-related role for WTAP-associated regulation by showing that a WTAP-associated mechanism promotes YTHDF2-dependent degradation of SYTL1 mRNA, thereby weakening natural killer cell–mediated antitumor activity. Collectively, these findings indicate that WTAP-associated mechanisms reinforce BCa progression through coordinated effects on glycolytic adaptation, ferroptosis resistance, and immune evasion.

### 5.2 m^6^A “erasers” in BCa

In BCa, the eraser layer is more functionally polarized than the writer layer, with mechanisms involving ALKBH5 more often showing tumor-suppressive consequences, whereas those involving FTO more often produce pro-tumor consequences (97–100).

ALKBH5-associated mechanisms are primarily linked to suppression of angiogenesis and metastatic dissemination in BCa. Xie et al (97). demonstrated that the lncRNA BLACAT3 is aberrantly upregulated in BCa and is associated with poor prognosis in muscle-invasive disease. Mechanistically, m^6^A modification stabilizes BLACAT3, whereas loss of ALKBH5 increases the m^6^A level and structural stability of BLACAT3. Stabilized BLACAT3 then recruits YBX3 into the nucleus, where the BLACAT3–YBX3 complex enhances NCF2 transcription and activates NF-κB signaling, thereby promoting angiogenesis and hematogenous metastasis. These findings support a model in which the ALKBH5/BLACAT3-associated mechanism restrains BCa progression by limiting m^6^A-dependent stabilization of BLACAT3 and its downstream pro-angiogenic and pro-metastatic program.

By contrast, the currently reported mechanisms involving FTO in BCa predominantly produce pro-tumor consequences. Previous studies (98, 99) showed that FTO-associated mechanisms promote BCa progression through the FTO/miR-576/CDK6 axis as well as the MALAT1/miR-384/MAL2 regulatory network. In addition, an FTO-associated mechanism contributes to metabolic adaptation by reducing the m^6^A level of PYCR1 mRNA, thereby enhancing its transcript stability and expression. Elevated PYCR1 further promotes BCa proliferation and invasion (100). Taken together, available evidence indicates that FTO-associated mechanisms in BCa predominantly yield pro-tumor consequences, with recurrent outputs involving cell-cycle progression, lncRNA-mediated survival signaling, and metabolic rewiring.

### 5.3 m^6^A “readers” in BCa

In BCa, the principal m^6^A readers include YTH domain family proteins and IGF2BP family members, which together regulate transcript stability, translational output, stemness, and pro-tumor signaling. Overall, the reader layer in BCa is centered on mechanisms involving YTHDF1, YTHDF3, and IGF2BP3, whose reported downstream programs more often produce pro-tumor consequences, whereas mechanisms involving YTHDC1 more often show tumor-suppressive consequences (101–106).

Among the currently described reader-associated mechanisms in BCa, those involving YTHDF1 are the most consistently linked to pro-tumor consequences. Le et al (101). demonstrated that the m^6^A–YTHDF1 axis promotes BCa progression by regulating GRIN2D and reshaping tumor metabolism, particularly aerobic glycolysis. Consistently, Zhu et al (102). reported that the METTL3/YTHDF1–RPN2–PI3K/AKT/mTOR cascade promotes BCa growth and migration. Together, these studies indicate that YTHDF1-associated mechanisms are major contributors linking m^6^A regulation to metabolic reprogramming, proliferative signaling, and migratory behavior in BCa.

Mechanisms involving YTHDF3 also contribute to malignant progression, particularly at the level of bladder cancer stemness. Qiu et al (103). reported that silencing YAP1 suppresses growth, invasion, migration, sphere formation, and stemness-associated protein expression in bladder cancer stem cells, while simultaneously reducing YTHDF3 and TGF-β1 and increasing SMAD7. Importantly, ectopic overexpression of YTHDF3 reverses these suppressive effects. These findings suggest that a YTHDF3-associated mechanism functions downstream of YAP1 to maintain bladder cancer stemness and tumorigenic potential, at least in part through TGF-β–associated signaling.

In contrast to the predominantly pro-tumor functional consequences linked to several cytoplasmic reader-associated mechanisms, YTHDC1-associated mechanisms in BCa more often show tumor-suppressive consequences in a nuclear regulatory context. Liu et al (104). identified the LINC01106/miR-3148/DAB1 axis as an inhibitory pathway in BCa progression and demonstrated that LINC01106 contains a functional m^6^A site recognized by YTHDC1. Both the expression level and m^6^A methylation of LINC01106 are reduced in BCa tissues, and low LINC01106 expression correlates with poor prognosis. Importantly, enforced methylation of LINC01106 using a CRISPR/dCas13b–METTL3–METTL14 system suppresses malignant phenotypes of BCa cells, whereas DAB1 knockdown reverses this effect. These observations indicate that YTHDC1-associated mechanisms mediate lncRNA-dependent tumor-suppressive consequences in BCa within a nuclear regulatory context.

Mechanisms involving IGF2BP family members also participate in BCa progression, mainly through regulation of RNA stability. Xie et al (106). reported that circPTPRA suppresses BCa proliferation, migration, and invasion by antagonizing IGF2BP1 activity. Mechanistically, circPTPRA directly binds IGF2BP1 and impairs its ability to stabilize pro-tumor transcripts such as MYC and FSCN1, while also partially blocking KH-domain recognition of m^6^A-modified RNA. Conversely, Huang et al (105). demonstrated that IGF2BP3 is highly expressed in BCa tissues and associated with poor prognosis. Mechanistically, an IGF2BP3-associated mechanism promotes BCa proliferation by activating JAK/STAT signaling, and pharmacological blockade of JAK/STAT attenuates this pro-tumor effect. Consequently, these findings suggest that mechanisms involving the IGF2BP family in BCa more often produce pro-tumor consequences overall, although the specific functional output remains dependent on the RNA substrate and regulatory context engaged in a given model.

In summary, current evidence indicates that m^6^A regulation in BCa is organized around a relatively coherent network centered mainly on mechanisms involving METTL3, WTAP, FTO, and YTHDF1, whose reported downstream programs recurrently produce pro-tumor consequences, with major phenotypic outputs involving proliferation, invasion and migration, stemness, metabolic rewiring, angiogenesis, and therapy-related adaptation. Representative m6A regulators and their associated mechanisms in BCa are summarized in **Table 3**. At the same time, mechanisms involving METTL14, ALKBH5, and YTHDC1 provide important tumor-suppressive counterweights within this framework. Thus, compared with the more sharply contradictory patterns seen in selected regulator-associated mechanisms in PCa and RCC, the functional landscape of m^6^A regulation in BCa appears more convergent, while still retaining limited context-dependent variation at the level of specific reader- and lncRNA-centered pathways (85–106).

**Table 3:** Representative m^6^A regulators and associated mechanisms in BCa.

m^6^A regulators	Roles in m^6^A	Molecular mechanism	Functional consequence of the mechanism in cancer	Ref
METTL3	Writer	METTL3/DGCR8/pri-miR-221/222	Promotes tumor proliferation	(85)
METTL3	Writer	METTL3/miR-146a-5p/Numb/NOTCH2	Promotes tumor progression	(86)
METTL3	Writer	METTL3/AFF4/NF-κB/MYC	Promotes tumor proliferation, invasion, and survival	(87)
METTL3	Writer	METTL3/AFF4/SOX2/MYC	Promotes tumor stemness and progression	(88)
METTL3/YTHDF2	Writer/Reader	METTL3/SETD7/KLF4/YTHDF2	Promotes tumor proliferation and metastasis	(89)
METTL3/YTHDF1	Writer/Reader	METTL3/CDCP1/YTHDF1	Promotes tumorigenesis	(90)
METTL3/YTHDF1	Writer/Reader	RBM15/METTL3/ENO1/YTHDF1/PCNA	Promotes tumor proliferation and growth	(91)
METTL3	Writer	METTL3/TEK/VEGF-A	Promotes tumor proliferation and angiogenesis	(92)
METTL14	Writer	METTL14/Notch1/tumor-initiating cells	Inhibits tumor progression and tumor-initiating potential	(93)
WTAP/IGF2BP2	Writer/Reader	WTAP/IGF2BP2/PIGT/GLUT1	Promotes tumor progression and glycolysis	(94)
WTAP/YTHDF1	Writer/Reader	WTAP/NRF2/YTHDF1	Promotes tumor progression	(95)
WTAP/YTHDF2	Writer/Reader	WTAP/YTHDF2/SYTL1	Promotes tumor progression and immune evasion	(96)
ALKBH5	Eraser	ALKBH5/BLACAT3/YBX3/NCF2/NF-κB	Inhibits tumor progression, angiogenesis, and metastasis	(97)
FTO	Eraser	FTO/miR-576/CDK6	Promotes tumor proliferation and invasion	(98)
FTO	Eraser	FTO/lnc-MALAT1/miR-384/MAL2	Promotes tumor progression and cell viability	(99)
FTO	Eraser	FTO/PYCR1	Promotes tumor proliferation, invasion, and metabolic adaptation	(100)
YTHDF1	Reader	YTHDF1/GRIN2D	Promotes tumor proliferation and aerobic glycolysis	(101)
YTHDF1	Reader	YTHDF1/RPN2/PI3K/AKT/mTOR	Promotes tumor proliferation and migration	(102)
YTHDF3	Reader	YAP1/YTHDF3/TGF-β-associated signaling/SMAD7	Promotes tumor stemness	(103)
YTHDC1	Reader	YTHDC1/LINC01106/miR-3148/DAB1	Inhibits tumor progression	(104)
IGF2BP3	Reader	IGF2BP3/JAK/STAT	Promotes tumor proliferation	(105)

## 6 m^6^A methylation regulators in renal cell carcinoma

Current evidence indicates that m^6^A regulation in RCC forms a structured but non-uniform regulatory landscape rather than a simple network in which individual regulators can be uniformly interpreted as pro-tumor or tumor-suppressive. Representative m6A regulators and their associated mechanisms in RCC are summarized in **Table 4**. Across the currently available literature, recurrent biological outputs mainly converge on tumor growth, metastatic behavior, metabolic reprogramming, autophagy-related adaptation, EMT-associated plasticity, and PI3K/AKT-related signaling (107–114, 127–129). Within this framework, mechanisms in the writer layer more often yield tumor-suppressive consequences in METTL3- and METTL14-associated settings, whereas those involving WTAP more consistently produce pro-tumor consequences (107–114, 127–129). The eraser layer is centered on mechanisms involving FTO and shows the greatest functional divergence, although the currently available evidence still leans overall toward pro-tumor consequences, whereas the currently described ALKBH5-associated mechanism is supported only by single-study evidence and also produces pro-tumor consequences (115–119, 130). By contrast, the reader layer shows the most directionally convergent pattern, being organized predominantly around mechanisms involving the IGF2BP family that more often produce pro-tumor consequences, with one clearly defined suppressive exception (120–126).

**Table 4:** Representative m^6^A regulators and associated mechanisms in RCC.

m^6^A regulators	Roles in m^6^A	Molecular mechanism	Functional consequence of the mechanism in cancer	Ref
METTL3	Writer	METTL3/HHLA2	Promotes tumor progression	(107)
METTL3/IGF2BP1	Writer/Reader	METTL3/IGF2BP1/ZHX2	Promotes tumor progression	(108)
METTL3	Writer	METTL3-associated circPTEN/GLUT1 axis	Inhibits tumor progression and resistance to mTOR inhibitors	(109)
METTL3/METTL14	Writer	VHL/METTL3-METTL14/PIK3R3/p85/PI3K/AKT	Inhibits tumor progression	(110)
METTL14/YTHDC1	Writer/Reader	METTL14/lnc-LSG1/ESRP2/YTHDC1	Inhibits tumor invasion and migration	(111)
METTL14/YTHDF2	Writer/Reader	METTL14/lnc-NEAT1/YTHDF2	Inhibits tumor progression and metastasis	(112)
METTL14/YTHDF2	Writer/Reader	METTL14/ITGB4/YTHDF2	Inhibits tumor progression and migration	(113)
METTL14	Writer	METTL14/PTEN/PI3K/AKT	Inhibits tumor progression and migration	(114)
ALKBH5	Eraser	ALKBH5/AURKB	Promotes tumor proliferation, migration, and invasion	(115)
FTO	Eraser	FTO/PGC-1α	Inhibits tumor progression	(116)
FTO/IGF2BP2	Eraser/Reader	FTO/IGF2BP2/SIK2/autophagy	Promotes tumor progression	(117)
FTO	Eraser	FTO/OGDHL/TFAP2A/FASN/ERK	Promotes tumor progression and lipid synthesis	(118)
FTO/YTHDF2	Eraser/Reader	FTO/PDK1/YTHDF2	Promotes tumor proliferation, migration, EMT-associated progression, and metastasis	(119)
IGF2BP1	Reader	IGF2BP1/LDHA	Promotes tumor energy metabolism	(120)
IGF2BP2	Reader	circ-TNPO3/IGF2BP2/SERPINH1	Inhibits tumor progression and migration	(121)
IGF2BP2	Reader	IGF2BP2/lnc-DUXAP9/PI3K/AKT	Promotes tumor proliferation, migration, and invasion	(122)
IGF2BP3	Reader	IGF2BP3/lnc-CDKN2B-AS1/NUF2	Promotes tumorigenesis and progression	(123)
IGF2BP3	Reader	lnc-DMDRMR/IGF2BP3/CDK4	Promotes tumorigenesis and progression	(124)
IGF2BP3	Reader	IGF2BP3/AGAP2-AS1/miR-9-5p/THBS2/PI3K/AKT	Promotes tumor progression and macrophage M2 polarization	(125)
IGF2BP3	Reader	IGF2BP3/circRARS	Promotes tumor progression and lipid accumulation	(126)

### 6.1 m^6^A “writers” in RCC

In RCC, the principal m^6^A writers include METTL3, METTL14, and WTAP. Overall, the current writer layer in RCC is weighted more toward mechanisms whose functional consequences are tumor-suppressive in METTL3- and METTL14-associated settings, whereas mechanisms involving WTAP are more consistently associated with malignant progression. Functionally, the reported outputs of RCC writers mainly converge on regulation of tumor growth, metastatic behavior, metabolic programs, and PI3K/AKT-related signaling (107–114, 127–129).

Among these regulators, METTL3 represents a writer whose associated mechanisms in RCC appear particularly sensitive to upstream and downstream molecular context. Current evidence does not support a simple uniform functional interpretation of METTL3-associated mechanisms. Rather, the currently available literature more often links METTL3-associated mechanisms to tumor-suppressive consequences, although two studies support distinct transcript-centered settings in which the downstream programs are pro-tumor. On the tumor-suppressive side of the currently described METTL3-associated mechanisms, Zhan et al (109). demonstrated that the circPTEN-related program inhibits clear cell renal cell carcinoma (ccRCC) progression and resistance to mTOR inhibitors by reducing methylation of the PTEN promoter and decreasing m^6^A modification of GLUT1 mRNA, thereby suppressing glycolytic reprogramming. Consistently, Lee et al (110). showed that in VHL-deficient ccRCC, VHL coordinates assembly of the METTL3/METTL14 writer complex, stabilizes PIK3R3 mRNA, promotes p85 ubiquitination, inhibits PI3K/AKT signaling, and ultimately suppresses tumor growth in both cellular and murine models. In addition, Gan et al (129). reported that enhancement of MUC15 expression and m^6^A modification of HRG mRNA using a dCas13b–M3M14 fusion protein significantly inhibits kidney cancer metastasis. In summary, these studies more often link METTL3-associated mechanisms in RCC to metabolism-restraining, signaling-suppressive, and metastasis-inhibitory programs.

At the same time, two studies provide counterevidence supporting pro-tumor consequences of METTL3-associated mechanisms with relatively complete mechanistic support. Zhu et al (107). reported that METTL3 is significantly upregulated in ccRCC tissues, that higher METTL3 expression is associated with poorer survival, and that METTL3 depletion suppresses cell viability, migration, and invasion. Mechanistically, this study combined expression analyses, loss-of-function assays, MeRIP-qPCR, and rescue experiments to show that an METTL3-associated mechanism promotes ccRCC progression by mediating m^6^A modification of HHLA2 mRNA, whereas HHLA2 overexpression reverses the inhibitory effects induced by METTL3 depletion. Similarly, Xiao et al (108). showed that METTL3-mediated m^6^A modification, together with IGF2BP1-dependent stabilization of ZHX2, promotes RCC progression. This model was supported by tissue and cellular expression analyses, gain- and loss-of-function experiments, apoptosis, invasion, and spheroid assays, as well as *in vivo* tumor formation and ZHX2-centered rescue experiments showing that ZHX2 overexpression attenuates the suppressive effects of METTL3 silencing. Notably, these reports supporting pro-tumor consequences converge on m^6^A-dependent activation or stabilization of pro-tumor transcript programs centered on HHLA2 and IGF2BP1/ZHX2. At present, METTL3-associated mechanisms in RCC are better regarded as predominantly linked to tumor-suppressive consequences, while notable pro-tumor counterevidence remains in selected transcript-centered settings, rather than supporting any uniform functional interpretation.

Compared with METTL3-associated mechanisms, those involving METTL14 in RCC show more directionally consistent tumor-suppressive consequences. Current studies repeatedly indicate that METTL14-associated mechanisms restrain RCC progression through coordinated suppression of metastasis-associated lncRNA programs, EMT-related behavior, and PI3K/AKT signaling. At the level of lncRNA-mediated regulation, Shen et al (111). demonstrated that silencing METTL14 significantly enhances RCC metastasis both *in vitro* and *in vivo*. Mechanistically, an METTL14-associated m^6^A mechanism acting on Lnc-LSG1 prevents its interaction with ESRP2, whereas recognition of m^6^A-modified Lnc-LSG1 by YTHDC1 disrupts this interaction and suppresses metastatic progression. Consistently, Liu et al (112). reported that an METTL14-associated mechanism suppresses Lnc-NEAT1_1 expression through YTHDF2-dependent RNA degradation, thereby inhibiting RCC progression and metastatic dissemination. Together, these findings support the view that METTL14-associated mechanisms exert tumor-suppressive consequences in the control of lncRNA-driven metastatic behavior. Beyond lncRNA regulation, METTL14-associated mechanisms also inhibit RCC progression through EMT- and PI3K/AKT-related pathways. In ccRCC, ITGB4 is significantly overexpressed and strongly associated with migration, invasion, EMT, poor prognosis, and elevated metastatic risk, whereas an METTL14-associated mechanism accelerates ITGB4 mRNA degradation via m^6^A modification in a YTHDF2-dependent manner, thereby reducing ITGB4 expression and attenuating metastatic potential (113). Similarly, Zhang et al (114). demonstrated that METTL14 is significantly downregulated in ccRCC and that its overexpression inhibits cell proliferation and migration while suppressing PI3K/AKT signaling through m^6^A-dependent stabilization of Pten mRNA. Taken together, current evidence indicates that METTL14-associated mechanisms in RCC relatively consistently yield tumor-suppressive consequences, with recurrent effects centered on restriction of lncRNA-mediated metastasis, EMT-associated plasticity, and PI3K/AKT signaling.

WTAP-associated mechanisms represent a different pattern within the RCC writer layer and are more consistently linked to malignant progression. The most direct evidence indicates that WTAP-associated mechanisms in RCC produce pro-tumor consequences. He et al. (127) reported that miR-501-3p is significantly downregulated in RCC cell lines and clinical specimens, and that enforced expression of miR-501-3p inhibits RCC cell proliferation by inducing G1-phase cell-cycle arrest. Mechanistically, WTAP was identified as a direct downstream target of miR-501-3p, and WTAP knockdown phenocopied the tumor-suppressive effects of miR-501-3p overexpression while reducing global m^6^A levels. These findings support a model in which WTAP-associated mechanisms exert direct pro-tumor consequences in RCC. In parallel, Ying et al (128). demonstrated that EGR2 promotes RCC tumorigenesis by transcriptionally upregulating IGF2BP family members, which in turn enhance the stability of S1PR3 mRNA through an m^6^A-dependent mechanism and activate PI3K/AKT signaling. Although this study is better interpreted as supporting a broader m^6^A-dependent pro-tumor context than as direct evidence for WTAP-associated mechanisms, it remains consistent with the pro-tumor writer-associated framework in RCC. In short, the currently available evidence more consistently indicates that WTAP-associated mechanisms in RCC yield pro-tumor consequences.

Collectively, current evidence suggests that the RCC writer layer is organized around METTL3- and METTL14-associated mechanisms that more often produce tumor-suppressive consequences, whereas mechanisms involving WTAP more consistently yield pro-tumor consequences. Within this framework, the principal point of non-uniformity lies in METTL3-associated regulation, whose reported effects extend from metabolic and signaling restraint to HHLA2- and IGF2BP1/ZHX2-centered pro-tumor programs. Overall, the recurrent outputs of RCC writers involve modulation of tumor growth, metastatic behavior, metabolic regulation, and PI3K/AKT signaling, with distinct directional tendencies across individual regulators (107–114, 127–129).

### 6.2 m^6^A “erasers” in RCC

In RCC, the currently reported m^6^A erasers mainly include FTO and ALKBH5. Overall, the eraser layer shows a non-uniform functional pattern, with FTO representing the main source of divergence, whereas ALKBH5 is supported only by single-study evidence. Even so, the currently available evidence remains weighted more toward pro-tumor consequences overall. Functionally, RCC erasers have mainly been linked to metabolic regulation, autophagy-related adaptation, proliferative and migratory signaling, and transcript stability control (115–119, 130).

Among these erasers, mechanisms involving FTO are the most extensively investigated in RCC and also show the greatest functional polarization. The currently available evidence supporting pro-tumor consequences converges mainly on three directions: autophagy restraint, metabolic rewiring, and AKT-related proliferative signaling. Xu et al (117). demonstrated that FTO is significantly elevated in ccRCC and that silencing FTO impairs tumor growth and metastasis, while pharmacologic inhibition of FTO with FB23–2 suppresses tumor growth in a patient-derived xenograft model. Mechanistically, this study showed that an FTO-associated mechanism regulates SIK2 mRNA stability through an IGF2BP2-dependent mechanism, thereby restraining autophagic flux and promoting tumorigenesis. In parallel, Shi et al (118). showed that an FTO-associated demethylation mechanism suppresses OGDHL expression in ccRCC, relieves inhibition of the TFAP2A/FASN axis, promotes lipid accumulation, and activates ERK signaling, thereby supporting proliferation, migration, invasion, and metastatic progression. Likewise, Shen et al (119). demonstrated that FTO is significantly upregulated in ccRCC and that FTO knockdown suppresses proliferation, migration, EMT-associated changes, tumor growth, and metastasis. Mechanistically, an FTO-associated mechanism reduced the m^6^A level of PDK1 mRNA, prevented YTHDF2-mediated degradation of the transcript, and thereby preserved PDK1 expression and downstream AKT phosphorylation. Overall, these studies more often link FTO-associated mechanisms to pro-tumor programs centered on autophagy inhibition, metabolic reprogramming, and AKT-linked malignant progression.

At the same time, available evidence also supports selected RCC contexts in which FTO-associated mechanisms produce tumor-suppressive consequences. Zhuang et al (116). provided the most direct suppressive evidence by showing that FTO is downregulated in ccRCC tissues, that low FTO expression correlates with greater tumor severity and poorer survival, and that the FTO/PGC-1α axis suppresses tumor growth. Mechanistically, FTO reduced m^6^A levels in PGC-1α mRNA, increased PGC-1α expression, restored mitochondrial activity, and induced oxidative stress and ROS accumulation, thereby restraining ccRCC progression. A second study provided indirect but still relevant supportive evidence for this suppressive side. Yang et al (130). showed that miR-155 directly targets the 3′-UTR of FTO, reduces FTO expression, and increases global m^6^A levels. Functionally, miR-155 overexpression promoted proliferation and reduced apoptosis, whereas restoration of FTO counteracted these effects *in vitro* and *in vivo*. Although this study is centered primarily on the pro-tumor action of miR-155 rather than on a standalone FTO suppressive mechanism, it nevertheless supports the view that loss of FTO can facilitate ccRCC progression. Taken together, the suppressive evidence indicates that FTO-associated mechanisms in RCC are not uniformly pro-tumor, although this side of the literature is currently narrower than the body of evidence supporting pro-tumor consequences.

Compared with the literature on FTO-associated mechanisms, ALKBH5 is represented only by a single RCC-focused study, and this study supports pro-tumor consequences in that specific model. Zhang et al (115). showed that ALKBH5 is significantly upregulated in RCC tissues and cell lines, and that higher ALKBH5 expression is associated with larger tumor size, higher TNM stage, and worse overall survival. Functionally, ALKBH5 overexpression promoted proliferation, colony formation, cell-cycle progression, migration, and invasion, whereas ALKBH5 depletion suppressed tumor growth in xenograft models. Mechanistically, ALKBH5 directly bound AURKB mRNA, enhanced its stability in an m^6^A-dependent manner, and sustained downstream proliferative output. The study further suggested that hypoxia-induced HIF signaling may reinforce this ALKBH5/AURKB axis. Since this is currently the only ALKBH5-focused study in RCC under discussion here, it is best interpreted as single-study evidence of pro-tumor consequences rather than as a basis for broader trend-level conclusions about ALKBH5-associated mechanisms in the RCC eraser landscape.

In summary, the current RCC eraser landscape is organized mainly around functionally divergent mechanisms involving FTO, while ALKBH5 remains limited to single-study evidence of pro-tumor consequences. The broader available literature more often links FTO-associated mechanisms to pro-tumor consequences, whereas the suppressive side is retained mainly in the FTO/PGC-1α model and is further supported indirectly by miR-155–mediated targeting of FTO. Thus, the RCC eraser layer is best understood as a partially polarized landscape in which mechanisms involving FTO represent the principal point of mechanistic divergence, while the overall currently available evidence still leans toward pro-tumor consequences (115–119, 130).

### 6.3 m^6^A “readers” in RCC

In RCC, the m^6^A reader landscape is dominated by mechanisms involving the IGF2BP family. Overall, the currently available evidence indicates that mechanisms involving IGF2BP1, IGF2BP2, and especially IGF2BP3 more often produce pro-tumor consequences, with recurrent effects on metabolic adaptation, proliferation, invasion, and PI3K/AKT-related signaling. This pattern is not entirely uniform, however, because one study identifies a distinct suppressive setting centered on the circ-TNPO3/IGF2BP2/SERPINH1 axis (120–126).

Among these readers, IGF2BP1 is most clearly linked to metabolic regulation in RCC. Yuan et al (120). demonstrated that an IGF2BP1-associated mechanism promotes aerobic glycolysis in ccRCC by stabilizing LDHA mRNA, thereby supporting tumor growth and metastatic dissemination. These findings indicate that IGF2BP1-associated mechanisms couple m^6^A-dependent transcript stabilization to metabolic adaptation and thereby produce pro-tumor consequences in RCC.

Mechanisms involving IGF2BP2 are also more often associated with malignant progression, particularly in lncRNA-driven pro-tumor signaling. Tan et al (122). reported that Lnc-DUXAP9 is significantly upregulated in ccRCC and binds to IGF2BP2 in an m^6^A-dependent manner, thereby enhancing its stability. Increased IGF2BP2-associated activity subsequently activates the PI3K/AKT pathway and upregulates Snail expression, promoting RCC cell proliferation, migration, and invasion. These findings support the view that IGF2BP2-associated mechanisms in RCC more often produce pro-tumor consequences, although a distinct suppressive setting has also been described.

Notably, however, IGF2BP2 also appears in a distinct suppressive setting that should be retained as an important counterexample within the RCC reader landscape. Pan et al (121). reported that circ-TNPO3 is significantly downregulated in ccRCC and that its reduced expression is associated with distant metastasis, advanced WHO/ISUP grade, and higher T stage. Functional experiments further showed that circ-TNPO3 inhibits ccRCC cell proliferation and migration *in vitro* and suppresses metastatic dissemination *in vivo*. Mechanistically, circ-TNPO3 directly binds IGF2BP2 and destabilizes SERPINH1 mRNA, thereby downregulating the SERPINH1–SNAIL/SLUG axis and restraining metastatic progression. Notably, this represents an unusual regulatory context, because IGF2BP2-associated regulation, which typically enhances target stability and expression, here participates in suppression of ccRCC migration through the circ-TNPO3/IGF2BP2/SERPINH1 axis. This finding is therefore best interpreted as a single suppressive exception in a distinct regulatory setting, rather than as evidence that IGF2BP2 is broadly tumor-suppressive in RCC.

Compared with mechanisms involving IGF2BP1 and IGF2BP2, those involving IGF2BP3 show the most concentrated pattern of pro-tumor consequences among currently reported m^6^A readers in RCC. Current studies repeatedly link IGF2BP3-associated mechanisms to programs governing proliferation, cell-cycle progression, invasion, and PI3K/AKT-related pro-tumor signaling. At the level of growth- and cell-cycle-related regulation, Xie et al (123). demonstrated that Lnc-CDKN2B-AS1, stabilized by IGF2BP3, drives RCC malignancy through epigenetic activation of NUF2 transcription, thereby promoting proliferation, migration, and invasion. Consistently, Gu et al (124). further showed that the lncRNA DMDRMR mediates regulation of m^6^A-modified CDK4 through IGF2BP3, driving ccRCC progression and supporting a role for IGF2BP3 in cell-cycle control. Beyond these growth-related programs, IGF2BP3-associated mechanisms also contribute to progression-associated signaling and tumor-supportive microenvironmental regulation. Xu et al (125). reported that AGAP2-AS1 stabilized by IGF2BP3 promotes macrophage M2 polarization in ccRCC through the miR-9-5p/THBS2/PI3K-Akt pathway, indicating that IGF2BP3-associated mechanisms can promote tumor progression through microenvironment-related signaling. More recently, Liu et al (126). showed that circRARS synergizes with IGF2BP3 to regulate RNA methylation recognition and promote RCC progression. In short, current evidence indicates that IGF2BP3-associated mechanisms are the most consistently linked to pro-tumor consequences among currently described readers in RCC.

To summarize, the current RCC reader landscape is organized predominantly around mechanisms involving the IGF2BP family that more often produce pro-tumor consequences. Within this framework, mechanisms involving IGF2BP1 are mainly linked to metabolic adaptation, those involving IGF2BP2 are more often associated with lncRNA-driven pro-tumor signaling but retain one notable suppressive exception in the circ-TNPO3/SERPINH1 setting, and IGF2BP3-associated mechanisms are the most consistently linked to pro-tumor consequences through recurrent effects on proliferation, invasion, cell-cycle progression, and PI3K/AKT-related pathways. Thus, the RCC reader layer is better understood as one in which the currently described mechanisms are predominantly pro-tumor, with one clearly defined suppressive counterexample, rather than as a broadly bidirectional regulatory landscape (120–126).

Collectively, the currently available evidence indicates that RCC is characterized by a structured but non-uniform m^6^A regulatory landscape. More specifically, mechanisms in the writer layer more often show tumor-suppressive consequences, the eraser layer is centered on functionally divergent mechanisms involving FTO but still leans overall toward pro-tumor consequences, and the reader layer shows the most directionally convergent pattern, with its currently described mechanisms more often producing pro-tumor consequences. What distinguishes RCC most clearly is that its m^6^A regulatory architecture is deeply embedded in metabolic conditioning, autophagy-related adaptation, and signaling plasticity, while the strength and direction of these effects vary asymmetrically across regulator-associated settings. In this context, the principal point of mechanistic tension lies in FTO-associated regulation, whereas the reader layer remains comparatively stable and the writer layer occupies an intermediate position between these two patterns. Overall, current RCC evidence supports a regulatory framework in which m^6^A-related programs repeatedly converge on tumor growth, metastatic behavior, EMT-associated plasticity, and PI3K/AKT-related signaling, but do so through markedly non-uniform regulatory routes (107–130).

## 7 Non-m^6^A methylation regulators in urological cancers

To date, m^6^A regulators remain the most extensively studied RNA methylation modulators in urological cancers. In contrast, investigations into non-m^6^A RNA methylation regulators are still at an early stage, and the available literature remains limited. Current evidence is derived predominantly from prostate cancer (PCa) and bladder cancer (BCa), while comparable data in renal cell carcinoma (RCC) remain sparse. Available studies suggest that a small number of m^5^C-, m^1^A-, and m^7^G-related regulators may participate in selected malignant phenotypes, but the overall mechanistic understanding of non-m^6^A pathways in urological malignancies remains fragmentary. Even so, these regulators represent an emerging frontier in epitranscriptomic research beyond the m^6^A-centered framework.

In prostate cancer (PCa), several non-m^6^A RNA methylation regulators have been functionally characterized. Zhang et al (131). reported that NSUN5-associated m^5^C writer activity is significantly upregulated in PCa. Activation of the CDK13/NSUN5/ACC1 axis promotes fatty acid synthesis and lipid accumulation, thereby contributing to PCa progression.

In addition, García-Vílchez et al (132). identified upregulated METTL1-associated m^7^G writer activity in PCa. As an m^7^G tRNA methyltransferase, METTL1 influences translational control and tumor biology. Its inhibition has been suggested to enhance antitumor immune responses and improve responsiveness to immunotherapy. Extending this line of evidence, Zhang et al (133). demonstrated that METTL1 is further upregulated in castration-resistant prostate cancer (CRPC), where it promotes disease progression by installing m^7^G modifications on CDK14 mRNA, thereby enhancing transcript stability and promoting CRPC progression.

In bladder cancer (BCa), non-m^6^A RNA methylation regulators have also been implicated in oncogenic processes. Monshaugen et al (134). identified an m^1^A writer-associated mechanism involving TRMT6/61A that is upregulated in BCa and enhances tumor cell survival by increasing resistance to cellular stress.

Beyond m^6^A regulation, Wang et al (135). demonstrated that m^5^C writer-associated regulation involving NSUN2 and m^5^C reader-associated regulation involving ALYREF are both significantly upregulated in urothelial carcinoma of the bladder. Mechanistically, ALYREF binds hypermethylated m^5^C sites on RABL6 and TK1 mRNAs via its K171 domain, promoting mRNA splicing and stability. ALYREF also binds m^5^C-modified sites on NSUN2 mRNA, thereby reinforcing NSUN2 expression and forming an m^5^C-dependent positive feedback loop.

Furthermore, Ying et al (136). reported that METTL1 is highly expressed in BCa and associated with poor prognosis. Through regulation of m^7^G tRNA modifications, METTL1 enhances translation of EGFR and EFEMP1, promoting BCa cell proliferation, migration, and invasion. These findings suggest that the METTL1–m^7^G–EGFR/EFEMP1 axis produces important pro-tumor consequences in bladder cancer.

## 8 Current evidence for the diagnostic relevance of RNA methylation regulators in urological cancers

Current evidence indicates that the diagnostic relevance of RNA methylation regulators in urological cancers remains at an early exploratory-to-translational stage (23, 137, 138). Across prostate, bladder, and renal malignancies, aberrant expression of selected regulators has been repeatedly observed; however, the supporting literature is still derived largely from tissue-based differential expression analyses, retrospective cohorts, and mechanism-oriented studies rather than from rigorous diagnostic validation (23, 137–140). As such, the field currently supports the concept of diagnostic-related candidacy more readily than true clinical diagnostic utility, and the available evidence is more appropriately viewed as exploratory to limited patient-derived support than as a clinically validated diagnostic framework (23, 137–140).

Among the major urological malignancies, PCa currently provides the most coherent diagnostic-related evidence for RNA methylation regulators (141–143). Several regulators, including METTL1 (133), METTL3 (65), METTL14 (68), YTHDF1 (80), and YTHDF2 (84), have been supported by paired tissues, tissue microarrays, or other forms of tissue-level validation in combination with public datasets, forming a modest yet comparatively consistent body of patient-derived diagnostic-related evidence (65, 68, 80, 133, 141–143). Taken together, the current PCa literature offers the strongest rationale for considering RNA methylation regulators as diagnostic-related candidates, although the evidence still remains well below the threshold required for clinical implementation (65, 68, 80, 133, 141–143).

In BCa, the study of RNA methylation regulators also remains at an early stage, but the literature is even more strongly weighted toward mechanistic interpretation (137–139, 144). Existing studies on regulators such as WTAP (139, 145) and YTHDF1 (102) primarily support their involvement in downstream molecular programs and tumor-associated biological processes, whereas METTL3 (85) has comparatively stronger tissue-level support and therefore represents one of the more plausible diagnostic-related markers in BCa (85, 102, 139, 145). Even so, the overall evidence base remains limited in both depth and consistency, and the currently available findings are better regarded as preliminary diagnostic-related signals than as the foundation of a mature diagnostic framework (85, 102, 139, 145).

By comparison, the diagnostic relevance of RNA methylation regulators in RCC is constrained not only by limited evidence, but also by pronounced biological heterogeneity (140, 146–150). Rather than converging on a stable expression pattern that might support diagnostic application, current studies more often point to context-dependent dysregulation, with METTL3 (140, 147) providing a particularly illustrative example. Its reported regulatory consequences in ccRCC vary across molecular settings and cannot yet be interpreted as a uniformly tumor-promoting or tumor-restraining signal (147). This degree of heterogeneity substantially weakens the current diagnostic interpretability of RNA methylation regulators in RCC and suggests that, at present, they are more appropriately viewed as indicators of biological perturbation than as reliable diagnostic classifiers (140, 146–150). Overall, the diagnostic value supported by the current RCC literature remains modest (140, 146–150).

## 9 Therapeutic and translational relevance of RNA methylation in urological cancers

### 9.1 Therapeutic relevance and current preclinical limitations

Current evidence supports a genuine link between RNA methylation and treatment response in urological cancers. What it does not yet support is the stronger claim that most of these regulators have become directly targetable therapeutic nodes. Across prostate, bladder, and renal cancers, the dominant pattern is repeated involvement in resistance, sensitization, and treatment-associated adaptation. The representative studies discussed below are sufficient to establish that therapeutic relevance, even though they do not capture the full mechanistic breadth of the field.

In prostate cancer, treatment-related work has focused mainly on docetaxel and androgen receptor signaling inhibitor (ARSI) resistance. Loss of UFL1 promotes enzalutamide resistance through METTL16-dependent regulation of EEF1A1. Because the *in vivo* evidence still relies on pre-engineered tumor cells rather than direct intervention against UFL1 or METTL16, this study is best regarded as preclinical genetic validation rather than therapeutic proof-of-concept (151). A similar limitation applies to circGLIS3, circRBM33, and the RBM15-IGF2BPs-associated docetaxel-resistance network, which are mechanistically informative but remain short of pharmacologic validation (60, 61, 77). The circQKI study should be interpreted even more cautiously. It suggests an m^6^A-circRNA layer in autophagy-driven docetaxel resistance, but it lacks patient validation and offers no *in vivo* therapeutic inhibition strategy; it therefore remains concept-level only (152).

In bladder cancer, the literature is denser, especially in cisplatin resistance. RNF220 is one of the more informative examples because it links chemotherapy resistance to PD-L1 upregulation and reduced CD8^+^ T-cell infiltration. Even so, the study stops short of direct *in vivo* targeting and is better regarded as strong mechanistic evidence with translational relevance than as therapeutic validation (153). YTHDF3 lactylation also supports treatment relevance, but again at the level of mechanistic and genetic validation rather than direct targetability (154). Metabolic adaptation is also represented in this setting. ALKBH5 loss promotes cisplatin resistance through CK2α-dependent glycolytic rewiring, and although CK2 inhibitors were effective *in vitro*, the absence of corresponding *in vivo* pharmacologic rescue keeps this study within preclinical genetic validation rather than true translational proof-of-concept (155). By contrast, the RBM15/IGFBP3 study incorporated genuine *in vivo* drug combination experiments to reverse cisplatin resistance, placing it among the few studies that reach limited pharmacologic proof-of-concept (156).

The renal cancer literature is smaller, but somewhat closer to translation. In clear cell renal cell carcinoma, the ERβ/circAHNAK/FMR1/ADAM17 study included true *in vivo* pharmacologic intervention with PHTPP and JG26 (157). CDK13-driven METTL16-ACLY signaling was likewise extended to *in vivo* testing with 1NM-PP1, providing one of the clearer examples of pathway-level pharmacologic validation in the field (158). The YY1/HDAC2-YTHDC1-ANXA1 axis in sunitinib resistance also stands out because CAY10683 was combined with sunitinib *in vivo*, giving the study firmer translational footing than most of the current literature (159). Even here, however, what has mainly been validated is the tractability of an upstream or parallel node rather than broad direct targetability of the core RNA methylation regulator itself.

### 9.2 The gap between biological plausibility and direct targetability

The central limitation of the field is not lack of mechanism, but lack of therapeutic depth. Patient-associated expression data, cell-based mechanistic work, and xenografts derived from pre-engineered cells can show that a pathway contributes to resistance. They do not, by themselves, establish realistic *in vivo* targetability. On that standard, studies such as UFL1/METTL16, circGLIS3, circRBM33, YTHDC1/PTEN, circ0008399/WTAP, ALKBH5/CK2α, and the RBM15-IGF2BPs-associated docetaxel-resistance network remain preclinical genetic validation, not direct therapeutic validation (60, 61, 77, 151, 155, 160, 161).

Targetability is also uneven across regulator classes. Writers and erasers are, in principle, more tractable because they contain catalytic pockets. Readers, circRNA-centered circuits, and RNA-protein interfaces are often harder to target directly. The present literature reflects that asymmetry. Some of the most interesting studies focus on reader- or circRNA-associated networks, yet remain confined to knockdown, overexpression, or rescue systems without a practical *in vivo* targeting modality (60, 61, 152, 154, 160). Even when a pharmacologic tool is introduced, it often acts on an upstream or downstream tractable node rather than on the RNA methylation regulator itself (61, 156–159).

Selectivity, toxicity, redundancy, and delivery remain underdeveloped as translational questions. The current urological literature rarely addresses therapeutic window, long-term safety, or tissue selectivity in a substantive way. Nor do most studies test whether resistance can escape through parallel pathways, even though the biology points strongly in that direction. Across the field, RNA methylation-associated treatment phenotypes repeatedly intersect with autophagy (152), checkpoint control (162), lactylation and chromatin repression (154, 158, 163), glycolysis (155), lipid metabolism (158), angiogenesis (157), and immune suppression (153, 163). This pattern argues against simple linear models of targetability. Moreover, for targets that resist conventional drugging—particularly circRNAs, scaffolding RNAs, and RNA-protein complexes—even where biological rationale is strong, direct targetability remains constrained by the absence of proven systemic and tumor-selective delivery strategies. Resolving this problem remains a prerequisite for meaningful translational progress. It also suggests that, even where a targetable vulnerability is identified, durable therapeutic benefit may depend on combinatorial rather than single-node intervention.

### 9.3 What current evidence supports, and what remains premature

A restrained conclusion is the correct one. RNA methylation regulators clearly participate in resistance and sensitization across prostate, bladder, and renal cancers. That point is well supported. What remains premature is the claim that most of these regulators are broadly ready for direct therapeutic targeting.

A small number of studies sit closer to translation than the rest. In bladder cancer, the RBM15/IGFBP3 study provides limited pharmacologic proof-of-concept because it includes genuine *in vivo* drug-based reversal of cisplatin resistance (156). In renal cancer, the ERβ/circAHNAK/FMR1/ADAM17, CDK13/METTL16/ACLY, and YTHDC1/ANXA1-sunitinib studies also move beyond engineered-cell xenografts by incorporating true *in vivo* drug intervention (156–159). These studies represent real progress, but they remain exceptions. More importantly, they generally validate tractable upstream or parallel nodes rather than direct therapeutic control of the RNA methylation regulator itself (156–159).

For the near term, two directions appear most realistic. The first is biomarker-guided stratification, although this still lacks treatment-response-annotated clinical validation in most settings (153, 159). The second is rational combination therapy, in which RNA methylation-associated vulnerabilities are incorporated into existing therapeutic backbones rather than pursued as stand-alone targets (156–159). Delivery barriers, particularly for targets that are not readily addressed by conventional drug design, are likely to define a longer-term frontier of the field. For now, the field supports therapeutic relevance, but not therapeutic readiness. Representative studies supporting the therapeutic relevance of RNA methylation-related regulators in urological cancers are summarized in **Table 5**. Bridging that gap will require direct *in vivo* pharmacologic validation, systematic selectivity and toxicity assessment, patient-response-annotated clinical evidence, and workable delivery strategies for targets that fall outside conventional drug design. Epitranscriptomic editing tools also deserve brief mention. A Cas13-directed methyltransferase system has enabled site-specific m^6^A installation in cellular RNAs and induced m^6^A-dependent changes in transcript abundance and alternative splicing, supporting its value primarily as a tool for functional interrogation rather than as current therapeutic evidence (164).

**Table 5:** Representative studies supporting the therapeutic relevance of RNA methylation-related regulators in urological cancers.

Cancer type	Target/pathway	Therapeutic context	Evidence type	What the study supports	Key limitation
Studies Approaching Pharmacologic Proof-of-Concept					
BCa	RBM15/IGFBP3	Cisplatin resistance	Limited pharmacologic proof-of-concept	*In vivo* reversal of cisplatin resistance with drug-based combination treatment	Validates pathway-level tractability rather than direct RBM15 targeting
ccRCC	ERβ/circAHNAK/FMR1/ADAM17	Angiogenesis-associated therapeutic relevance	Limited pharmacologic proof-of-concept	*In vivo* suppression of tumor growth and angiogenesis through pathway-level intervention	Drug-based validation targets tractable upstream/parallel nodes rather than the core regulator
ccRCC	CDK13/METTL16/ACLY	Lipid metabolism-associated therapeutic relevance	Limited pharmacologic proof-of-concept	*In vivo* suppression of a METTL16-dependent metabolic program	Pharmacologic intervention targets CDK13 rather than METTL16 directly
ccRCC	YY1/HDAC2/YTHDC1/ANXA1	Sunitinib resistance	Limited pharmacologic proof-of-concept	Pathway-level modulation of sunitinib response with *in vivo* drug intervention	Rescue occurs through HDAC2-targeting rather than direct YTHDC1 targeting
Representative Studies Supported Mainly by Preclinical Validation					
PCa	UFL1/METTL16/EEF1A1	Enzalutamide resistance	Preclinical genetic validation	METTL16-dependent control of enzalutamide response	Pre-engineered xenografts only; no direct *in vivo* UFL1/METTL16 targeting
BCa	RNF220/PDE10A	Cisplatin resistance; immune-evasive relevance	Preclinical mechanistic validation	Links cisplatin resistance to PD-L1 upregulation and reduced CD8^+^ T-cell infiltration	No direct *in vivo* targeting; patient evidence remains correlative
BCa	YTHDF3 (lactylation)/KDM6B	Cisplatin resistance	Preclinical genetic validation	Lactylation-associated m^6^A regulation in cisplatin resistance	Mechanistic evidence only; no *in vivo* drug-based targeting of the core axis
BCa	ALKBH5/CK2α/glycolysis	Cisplatin resistance	Preclinical genetic validation	Glycolytic rewiring associated with cisplatin resistance	*In vitro* pharmacologic rescue only; no *in vivo* drug-based validation

## 10 Current evidence for the immunological relevance of RNA methylation in urological cancers

Immunotherapy has become an important component of systemic treatment for selected urological malignancies, although its clinical impact varies substantially across tumor types. In metastatic urothelial carcinoma, long-term follow-up from pembrolizumab studies has demonstrated durable efficacy with no new safety signals, and final analysis of IMvigor210 likewise showed clinically meaningful activity with durable responses in a subset of patients receiving atezolizumab monotherapy (165, 166). In clear-cell renal cell carcinoma, the disease has been described as one of the most immune-infiltrated tumors in pan-cancer comparisons, and in clear-cell renal cell carcinoma several immune checkpoint blockade regimens have emerged as efficacious options that are now considered standards in treatment-naïve and pre-treated settings (167). By contrast, prostate cancer is widely regarded as an immune-cold malignancy, and the overall benefit from recent immunotherapeutic advances has remained limited in most patients, consistent with a predominantly immunosuppressive tumor immune microenvironment and generally poor responses to immune checkpoint blockade (168). This marked intertumoral heterogeneity suggests that additional regulatory layers may shape immune context and treatment responsiveness across urological cancers. In this setting, RNA methylation has emerged as a plausible regulatory layer linking tumor biology to immune-cell infiltration, immune dysregulation, and potentially therapeutic responsiveness, because accumulating evidence indicates that RNA methylation influences the tumor microenvironment, immune cells, immune factors, and immune evasion, with potential implications for immunotherapy (169). The following sections therefore examine how RNA methylation may shape immune infiltration states, immune evasion, antigen-presentation-related processes, and, where evidence is available, responses to immune checkpoint blockade in urological cancers.

### 10.1 RNA methylation and tumor immune microenvironment remodeling

At the level of the tumor immune microenvironment, recent subtype- and score-based analyses indicate that RNA methylation is associated with distinct immune infiltration states and broader heterogeneity across urological cancers. In bladder cancer, m^6^A-based subtype classification has identified biologically distinct groups with markedly different microenvironmental features, with the poorer-prognosis subtype showing higher stromal and inhibitory immune components together with lower computationally predicted responsiveness to immunotherapy (170). Similarly, the m^5^C reader ALYREF has also been linked to the bladder cancer immune microenvironment, particularly through its association with macrophage infiltration and its potential prognostic value (171). In prostate cancer, distinct m^6^A regulation patterns likewise correspond to different tumor immune microenvironment characteristics, and the poor-prognosis cluster was characterized by higher intratumoral heterogeneity together with a skewed immune infiltrative profile, including increased Th2-cell infiltration and lower infiltration of Th17 cells and M1/M2 macrophages (172). In clear cell renal cell carcinoma, comprehensive analyses of m^6^A modification patterns have similarly revealed biologically meaningful immune phenotypes, with m^6^A cluster 1 and the low-m^6^A-score group showing a hot and inflammatory tumor microenvironment characterized by increased cytotoxic immune-cell infiltration, higher checkpoint expression, and a phenotype that may be more sensitive to immunotherapy, whereas m^6^A cluster 2 and the high-m^6^A-score group display a more non-inflammatory phenotype (173).

### 10.2 RNA methylation and immune evasion/immunosuppressive programming

Beyond its association with immune infiltration patterns, RNA methylation may also facilitate immune escape in urological cancers through several mechanisms, although the current evidence is strongest in bladder cancer and remains more preliminary in prostate cancer. In bladder cancer, the JNK/METTL3 axis enhances PD-L1 expression by increasing m^6^A modification of PD-L1 mRNA and its IGF2BP1-mediated stabilization, thereby promoting resistance to CD8^+^ T-cell cytotoxicity and tumor immune escape (174). METTL3 has also been reported to remodel the bladder cancer microenvironment by increasing CXCL5 and reducing CCL5, thereby promoting MDSC recruitment, limiting CD8^+^ T-cell infiltration, and supporting an immunosuppressive state (175). In parallel, YTHDF2 suppresses RIG-I-mediated innate immune signaling by promoting m^6^A-dependent degradation of DDX58 mRNA, thereby facilitating immune evasion, limiting CD8^+^ T-cell recruitment, and reducing responsiveness to intravesical bacillus Calmette–Guérin (BCG) immunotherapy (176). Supporting this broader concept, prostate cancer data also suggest an immunosuppressive role for RNA methylation regulators: HNRNPC has been linked to an immune “cold” phenotype and a suppressive tumor microenvironment marked by increased Treg infiltration and reduced effector CD8^+^ T-cell features (177). Collectively, these studies indicate that RNA methylation may promote immune evasion by reshaping the tumor microenvironment and dampening antitumor immunity through mechanisms including checkpoint-associated regulation, attenuation of innate immune signaling, and reinforcement of suppressive immune states, thereby providing a mechanistic context for considering its potential effects on antigen presentation and immunotherapy responsiveness.

### 10.3 RNA methylation and antigen presentation

Compared with the evidence for immune infiltration and immune evasion, direct evidence linking RNA methylation to antigen presentation in urological cancers remains scarce. Current discussion therefore relies largely on mechanistic insights from general tumor immunology, particularly those related to dendritic cell-mediated antigen cross-presentation, which is critical for tumor-specific CD8^+^ T-cell priming within the cancer-immunity cycle (178, 179). More specifically, dendritic cells capture and process exogenous tumor antigens for presentation on major histocompatibility complex class I molecules, thereby enabling effective CD8^+^ T-cell activation against tumors (179). In this context, a key mechanistic study in non-urological tumor models demonstrated that the m^6^A reader YTHDF1 restrains antitumor immunity in dendritic cells by limiting cross-presentation of tumor antigens and the subsequent cross-priming of CD8^+^ T cells *in vivo (*180). Mechanistically, YTHDF1 recognizes m^6^A-marked transcripts encoding lysosomal proteases and promotes their translation in dendritic cells (180). Because efficient cross-presentation depends in part on limiting excessive lysosomal degradation and preserving antigenic material, this mechanism provides a biologically plausible explanation for how YTHDF1 constrains cross-presentation capacity (179, 180). In these models, YTHDF1 deficiency increased antigen-specific CD8^+^ T-cell priming and enhanced the therapeutic efficacy of PD-L1 blockade, further supporting the immunological relevance of this pathway (180). Although these findings were not generated in urological tumor models, they provide a plausible mechanistic framework for the possibility that RNA methylation may influence antitumor immunity in part through the regulation of antigen cross-presentation (169, 180). Therefore, in urological cancers, the relationship between RNA methylation and antigen presentation should currently be regarded as mechanistically plausible and biologically supported, but still lacking direct validation in urological tumor models.

### 10.4 RNA methylation and responses to immune checkpoint blockade

From a translational perspective, retrospective, computational, and preclinical studies suggest that RNA methylation-related programs may contribute to heterogeneous responses to immune checkpoint blockade in urological cancers, although the strength of evidence remains uneven across tumor types and study designs. In advanced urothelial carcinoma, retrospective analysis of the IMvigor210 cohort showed that several m^6^A regulators were differentially expressed between responders and non-responders to atezolizumab, and a nomogram integrating an m^6^A-related gene signature with tumor mutational burden and PD-L1 expression on immune cells showed potential predictive value for treatment response in a retrospective modeling framework (181). In parallel, m^6^A-based subtype stratification studies in bladder cancer have suggested that tumors with more stromal enrichment and a more suppressive immune context may show lower computationally predicted immunotherapy responsiveness, indicating that RNA methylation-associated tumor states may contribute to heterogeneity in treatment sensitivity, although this evidence remains indirect (170). Mechanistic studies provide additional biological support for this association. Consistent with its immunosuppressive role in bladder cancer, silencing or pharmacologic inhibition of METTL3 enhanced anti-PD-1 efficacy and increased CD8^+^ T-cell infiltration in preclinical models (175). Although derived from a non-checkpoint immunotherapy setting, YTHDF2-mediated suppression of RIG-I signaling in bladder cancer further suggests that RNA methylation may modulate immunotherapeutic benefit through innate immune mechanisms (176). In ccRCC, two transcriptome-based studies linked m^6^A-related patterns and m^6^A scores to distinct immune phenotypes, prognosis, and putative differences in immunotherapy sensitivity; however, much of this evidence remains based on retrospective stratification and computational inference, including one study that associated m^6^A score-related features with survival differences in an anti-PD-1-treated advanced ccRCC cohort (173, 182). Collectively, these findings suggest a potentially clinically relevant connection between RNA methylation and immunotherapy responsiveness in urological cancers, but current evidence remains uneven across tumor types and still derives predominantly from retrospective modeling, computational prediction, and regulator-specific preclinical studies rather than prospective clinical validation.

### 10.5 Summary and perspective: evidence hierarchy, translational relevance, and current limitations

Overall, accumulating evidence suggests that RNA methylation is linked to multiple dimensions of antitumor immunity in urological cancers, although the strength of support remains uneven across tumor types and study designs. Taken together, current evidence most consistently supports an association between RNA methylation and distinct immune infiltration patterns as well as broader immunoregulatory microenvironmental states, as indicated by subtype- and score-based studies across bladder, prostate, and renal cancers, together with regulator-specific analyses in bladder cancer (170–173). Selected mechanistic studies further support a contribution to immune evasion, including METTL3-dependent PD-L1 regulation, METTL3-driven suppressive chemokine remodeling, YTHDF2-mediated attenuation of innate immune sensing, and HNRNPC-associated Treg-skewed phenotypes (174–177). By comparison, associations with immune checkpoint blockade response are of potential clinical relevance but remain supported by a more heterogeneous evidence base that includes retrospective cohort modeling, computational stratification, and selected preclinical studies, with broader external validation and prospective clinical confirmation still limited (181, 182). Direct support is currently most limited for antigen presentation in urological cancers, where available evidence remains largely indirect and is derived primarily from general cancer-immunity-cycle frameworks and non-urological dendritic-cell mechanisms such as the YTHDF1 cross-presentation axis (178, 180).

Accordingly, the field is presently limited by heterogeneity in evidence type, frequent reliance on transcriptomic inference, and a current emphasis on regulator-level associations rather than direct modification-site-resolved validation. Although clinically annotated immunotherapy cohorts in metastatic urothelial carcinoma are beginning to support more refined response modeling, RNA methylation-based predictors still require broader external validation and stronger disease-context-specific confirmation before they can be regarded as robust translational tools (181, 183). Because disease-relevant RNA methylation analysis will likely depend on the broader maturation of epitranscriptomic technologies, recent advances in single-cell epitranscriptomic profiling and in quantitative, high-resolution mapping of RNA modifications begin to provide a methodological basis for this transition, although important technical challenges remain (55, 184). Meanwhile, emerging spatial epitranscriptomic strategies, although not yet established in urological cancers or RNA methylation-focused settings, support the feasibility of integrating transcriptomic, epitranscriptomic, and microenvironmental information within defined tumor microniches (185). Together with growing recognition that dendritic-cell dysfunction and impaired antigen presentation are important components of tumor immune escape (186), these developments point to the importance of prioritizing disease-specific validation in bladder, renal, and prostate cancers and of more directly linking RNA methylation states with clinically annotated immunotherapy outcomes, including response, resistance, and rational combination strategies.

## 11 Conclusion

RNA methylation has emerged as an important post-transcriptional regulatory layer in urological cancers (23, 24). Importantly, the biological effects of RNA methylation are determined not by chemical marks alone, but by coordinated regulatory proteins that install, remove, and interpret these modifications (17, 28–30). Among currently studied RNA modifications, m (7)A provides the most mature mechanistic framework, whereas non-m^6^A regulators remain comparatively less well characterized (16, 24, 25, 31). Within this framework, RNA methylation-associated regulation has been increasingly linked to tumor initiation and progression in urological cancers (19, 23–25).

Collectively, RNA methylation regulators influence the fate and function of diverse RNA species, including mRNA, tRNA, miRNA, circRNA, and lncRNA, and converge on major malignant phenotypes in urological cancers, including proliferation, invasion and migration, angiogenesis, drug resistance, autophagy, and immune evasion (24, 25, 28, 29). These observations support RNA methylation as a biologically important regulatory framework for understanding how post-transcriptional control contributes to urological tumor development and treatment-related adaptation (23, 24).

Substantial progress has been made in defining the role of RNA methylation, particularly m^6^A, in urological cancers. However, important gaps remain. Although research on m^6^A regulators has expanded considerably, investigations into non-m^6^A RNA methylation regulators are still at an early stage, and the available literature remains limited (24, 25, 31, 32, 131, 132, 134, 137). In addition, relatively few non-m^6^A “writers,” “erasers,” and “readers” have been clearly characterized, and their mechanistic roles remain insufficiently resolved (24, 25, 131–136). As a result, the broader regulatory network of RNA methylation in urological cancers is still incomplete, which constrains systematic biological interpretation and slows translational progress toward clinical application.

At the same time, accumulating evidence increasingly supports the diagnostic-related, prognostic, immunological, and therapeutic relevance of RNA methylation regulators in urological malignancies (137–141, 154–158, 169–174). Even so, current advances support biological and translational relevance more readily than clinically mature application. Future progress will depend not simply on identifying additional regulators, but on achieving higher-resolution epitranscriptomic interrogation, disease-context-specific mechanistic validation, and more rigorous translational testing for selectivity, safety, delivery feasibility, and direct *in vivo* pharmacologic relevance (22, 23, 48, 49, 55, 164, 184, 185).
